# Polarized neural responses to political narratives are sensitive to small variations in self-reported political perspectives

**DOI:** 10.1016/j.isci.2025.114268

**Published:** 2025-11-27

**Authors:** Niloufar Zebarjadi, Annika Kluge, Enrico Glerean, Matilde Tassinari, Iiro P. Jääskeläinen, Inga Jasinskaja-Lahti, Jonathan Levy

**Affiliations:** 1 Department of Neuroscience and Biomedical Engineering, School of Science, Aalto University, Espoo, Finland; 2 Faculty of Social Sciences, University of Helsinki, Helsinki, Finland; 3 Department of Criminology, Bar-Ilan University, Ramat Gan, Israel; 4 Gonda Brain Research Center, Bar-Ilan University, Ramat Gan, Israel

**Keywords:** Health sciences, Neuroscience, Social sciences

## Abstract

In an increasingly polarized and divided world, people often interpret new information through an ideologically biased lens (e.g., confirmation bias). Recent studies in the emerging field of political neuroscience report the phenomenon of “neural polarization”: cerebral activity that is shared (synchronized) between individuals holding similar political perspectives – but not between those holding dissimilar perspectives. Here, we extend this literature by testing for neural polarization between people with subtly different ideologies. Using functional magnetic resonance imaging (fMRI) while individuals (*n* = 40) listened to narratives about immigration in Finland, we observe neural polarization between more and slightly less immigration-supportive individuals in widespread neural areas, similar to the areas reported in previous studies of neural polarization. The findings extend current knowledge by revealing that neural polarization arises even when self-reported ideological perspectives differ only slightly. Together, these results shed light on how political information is interpreted and processed in the brain.

## Highlights

• Neural polarization occurs even with subtle ideological differences
• Widespread brain regions show polarized activity
• Neural polarization occurs both for congruent and incongruent ideological narratives
• The study sheds light on how confirmation bias is reflected through polarized brain activity

## Introduction

There is a worrying trend of increasing political and societal polarization across the world^1^ with pernicious effects for democracy.^2^ Polarization manifests as a clear distancing between “us” and “them,” for example, between conservatives and liberals, commonly researched in the USA^3^ but also clearly observable in many European countries.^4^ While Finland with its multiparty system exhibits relatively low societal affective polarization compared to other Western countries,^4^ polarization is still on the rise^5^^,^^6^ with climate and immigration topics at the center of debate.^7^^,^^8^^,^^9^^,^^10^ People tend to develop an ideological bias and interpret new information through a lens of their pre-existing beliefs so that both congruent and incongruent new information increase the political polarization.^11^^,^^12^^,^^13^^,^^14^ This phenomenon makes it increasingly difficult to find political consensus.

Political neuroscience has risen to interest due to its potential in explaining the origins of ideological bias.^15^ Some research shows that political attitudes bias perception already at sensory level.^16^^,^^17^^,^^18^ Recent neuropolitical research has focused on naturalistic stimuli.^19^^,^^20^^,^^21^^,^^22^^,^^23^ Van Baar et al. found that ideological similarity drives neural synchrony.^22^ Broom et al. showed that the more polarized participants were, the higher the neural synchrony.^23^ Leong et al.^21^ found differences in cognitive mechanisms, namely dorsomedial prefrontal cortex (DMPFC) activity between conservative- and liberal-leaning participants in the USA while watching the same immigration-policy related video clips. The neural polarization was tied to attitude divergence. De Bruin and colleagues^19^ found a link between polarized semantic representations of political concepts and polarized interpretation of naturalistic political content in the USA, especially in the amygdala on the immigration topic. Katabi et al.^20^ revealed ideology-dependent activations in higher-order regions as well as in early sensory, motor, and somatosensory regions while watching campaign ads and political speeches in Israel. In the last three studies we described, the brain responses predicted political orientation.^19^^,^^20^^,^^21^

While Leong and de Bruin did not differentiate the stimuli based on their political message, Katabi et al. also separately analyzed right- and left-wing political content and mapped brain regions with differences in activation between ideological groups for each video type separately. They found that only the right-wing individuals’ sensorimotor cortex was activated in processing right-wing content.

We set forth to study the differently polarized context of Finland. Based on earlier findings described here, we chose immigration as our topic. To attain strict experimental control while using naturalistic stimuli, we created balanced (similar length and content) pro- and anti-immigration audio to directly compare the neural responses to differently polarized narratives (see supplemental information, SI) and recorded fMRI data from 48 individuals while they listened to those narratives. Our study goals were preregistered [^24^, https://osf.io/9kjzy; full details in SI at Preregistration] and read as follows: we investigate whether identical information is interpreted differently depending on the worldview, or in other words, how issue attitudes bias the processing of immigration-related narratives.

Our experimental strategy relied on the successful strategy set forth in the previous studies investigating neural polarization^19^^,^^20^^,^^21^ and, in particular, that of the pioneering study by Leong and colleagues.^21^ This strategy was pre-registered and additionally detailed in the STAR Methods section. It can be summarized briefly as follows: First, participants perform a rating task on immigration narratives, and based on the ratings, we perform a median split to obtain two groups: one that is more supportive toward immigration, and one that is less supportive. Second, we perform computations on data that is shared between individuals with similar political attitudes (e.g., within the cohort which is more supportive of immigration) but not between individuals with dissimilar political attitudes (e.g., between the cohort which is more supportive of immigration and the cohort which is less supportive). This computation reflects the meaning of the “neural polarization” concept. Specifically, to localize in neural polarization, for each participant, a whole brain voxel-wise “within-group ISC (inter-subject correlation)” is computed with the neural activity of all other participants with similar political attitudes, and a “between-group ISC” with the participants who have dissimilar political attitudes. The difference between within-group and between-group ISC reflects the measure of neural polarization as outlined above. In other words, neural polarization is computed by highlighting voxels in the brain where within-group ISC is greater than between-group ISC.

We therefore raise three pre-registered hypotheses: (1) We will find brain region(s) that reflect the difference between the two groups of participants with different immigration support levels in processing immigration-related narratives. In addition, we examine whether the differences depend on the type of narrative, thus exploring (2) whether pro- and anti-immigration stimuli influence the results of our first hypothesis differently. Last, we will investigate (3) whether these neural differences are connected to behavioral self-reports.

## Results

### Immigration attitude score tied to multiple affective self-reports

**Figure 1:**
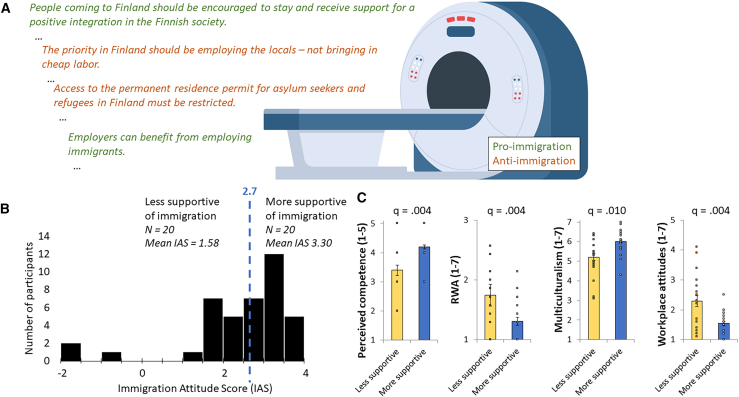
Experimental design and group division (A) Participants listened to pro- (written in green) and anti-immigration written (written in orange) narrative during an fMRI recording. After each statement, they rated their agreement with the statement on a scale of 1–5. The statements were presented in Finnish and are rephrased here in English. MRI image: macrovector/Freepik.com. (B) Distribution of immigration attitude scores. Immigration attitude score was calculated based on the self-rated agreement levels. The median score on a scale of −4 to 4 was 2.7, based on which the participants were divided into less and more immigration supportive (*N* = 20 in both groups). (C) Explicit attitude comparisons in less and more supportive participant groups. These graphs depict the group averages for the scales where there was a significant between-group difference: perceived competence (scale 1–5 where 5 means very competent), RWA (1–7, where 7 means very right-wing), Multiculturalism (1–7 where 7 means very supportive of multiculturalism), and Workplace attitudes (1–7 where 7 means extremely discriminatory attitudes). The q-values mark the FDR-corrected significance values of the between-groups t-tests. Data are represented as mean ± SD.

The two groups of participants were significantly different (FDR-corrected) on multiple explicit scales (Figure 1C): competence (t(38) = 3.487, q = 0.004, Cohen’s d = 0.103), RWA (t(38) = −3.636, q = 0.004, Cohen’s d = −1.150), multiculturalism (t(38) = 3.119, q = 0.010, Cohen’s d = 0.986), and discriminatory workplace attitudes (t(38) = 3.119, q = 0.010, Cohen’s d = 0.986). The rest of the self-reported attitudes did not significantly differ between the two groups (q > 0.087). All group averages and comparisons can be found in Table S1. Politically, the average participant in the more supportive group was leftist [average score 4.8, 95% CI [4.25, 5.35] on a score from 1 to 7, significantly differing from centrist (*p* = 0.012)] and in the less supportive group centrist [average score 4.4, 95% CI [3.86, 3.83], not significantly different from centrist (*p* = 0.185)].

Next, we were interested in how the Immigration Attitude Score (IAS) depends on these explicit attitudes and found a significant linear dependency (F(13) = 5.050, *p* < 0.001∗∗, R^2^_adj_ = 0.574, coefficients in Table S2). We separately tested which measures drive this relationship and calculated correlations with IAS. We found significant (FDR-corrected) correlations with empathic emotions (R = 0.539∗∗, q = 0.003), feeling thermometer (R = 0.399∗, q = 0.024), competence (R = 0.385∗, q = 0.026), perceived threat (R = −0.409∗∗, q = 0.023), multiculturalism (R = 0.663∗∗, q = 0.003), group superiority (R = −0.547∗∗, q = 0.003) and discriminatory workplace attitudes (R = −0.743∗∗, q = 0.003), validating the IAS and tying it to multiple affective explicit scales. The IAS was also significantly correlated with self-reported political inclination, affirming the connection to politics and allowing us to compare our results to earlier political research (R = 0.321∗, *p* = 0.044, no FDR-correction). The rest of the correlations were not statistically significant (q > 0.071, all correlations in Table S3).

### Analyzing all narratives together reveals between-group differences in neural activations

To address the first hypothesis, that is, to find brain regions that activate differently in the less and more immigration supportive participant groups while processing immigration-related stimuli regardless of the stimuli type, we searched for voxels where the activity time course was more similar within-group than it was between the groups (also referred to as within-between contrasts, full map of R value differences in NeuroVault https://identifiers.org/neurovault.image:867871). Two neural areas of significant difference (d stands for difference in R-values) emerged (Figure S1): left premotor cortex (MNI -50 6–2, d = 0.089, *p* = 0.002) and left dorsolateral prefrontal cortex (dlPFC) (MNI -40 38 38, d = 0.067, *p* < 0.001). We have detected brain regions that are processing immigration-related stimuli differently based on immigration support levels, thus we can reject the first null-hypothesis. The within-between contrasts were not significantly different in the two groups (t(38) = 0.738, *p* = 0.465 and t(38) = −0.628, *p* = 0.534, respectively), but if investigated separately, the premotor contrast was significant only in the more supportive group (t(19) = 3.077, *p* = 0.006 vs. t(19) = 1.353, *p* = 0.192), and the dlPFC only in the less supportive group (t(19) = 3.819, *p* = 0.001 vs. t(19) = 1.871, *p* = 0.077). The lack of significance in separate groups could be due to reduced statistical power when participants were divided into two.

### Different narrative types elicit different neural responses

**Figure 2:**
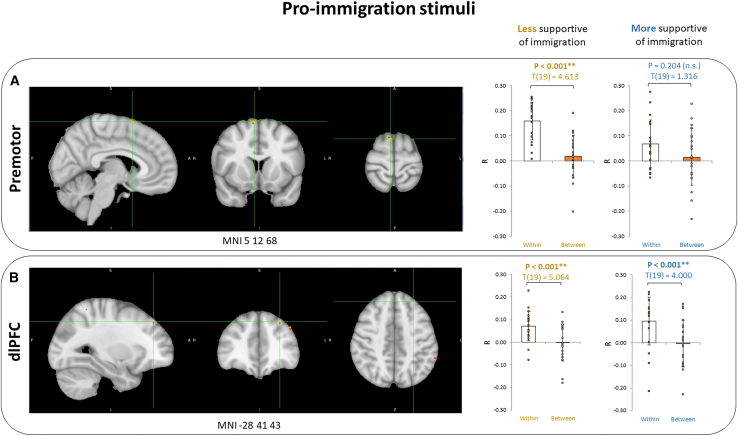
Pro-immigration stimuli Within-between contrast peaks in the (A) Premotor and in the (B) dlPFC, in response to pro-immigration stimuli and the contrasts separately in the groups of less immigration supportive and more immigration supportive participants. Data are represented as mean ± SD. Asterisks indicate statistical significance of *p* < 0.001 for the within-between contrast.

Next, to address the second hypothesis, we wanted to see whether different contrasts appear depending on the narrative type (anti- or pro-immigration), so we conducted a similar analysis for pro- and anti-immigration narratives separately. For pro-immigration stimuli (full map of R value differences in NeuroVault https://identifiers.org/neurovault.image:867872), we found two significant within-between group contrasts (Figure 2): in right premotor cortex (MNI 5 12 68, d = 0.097, *p* < 0.005), and in left dlPFC (MNI -28 41 43, d = 0.086, *p* <0 .001). Again, the contrasts were not significantly different in the less and more immigration supportive groups (t(38) = −1.749, *p* = 0.088 and t(38) = 0.869, *p* = 0.390, respectively). When investigated separately, the right premotor cortex contrast was only significant in the less supportive group (t(19) = 4.613, *p* < 0.001 vs. t(19) = 1.316, *p* = 0.204). The dlPFC contrast was significant in both groups (t(19) = 4.000, *p* < 0.001 for more supportive and t(19) = 5.064, *p* < 0.001 for less supportive participants). Thus, the findings behind pro-immigration stimuli were very similar to the findings of all stimuli investigated together, and again, the lack of significance in separate groups could be due to reduced statistical power.

**Figure 3:**
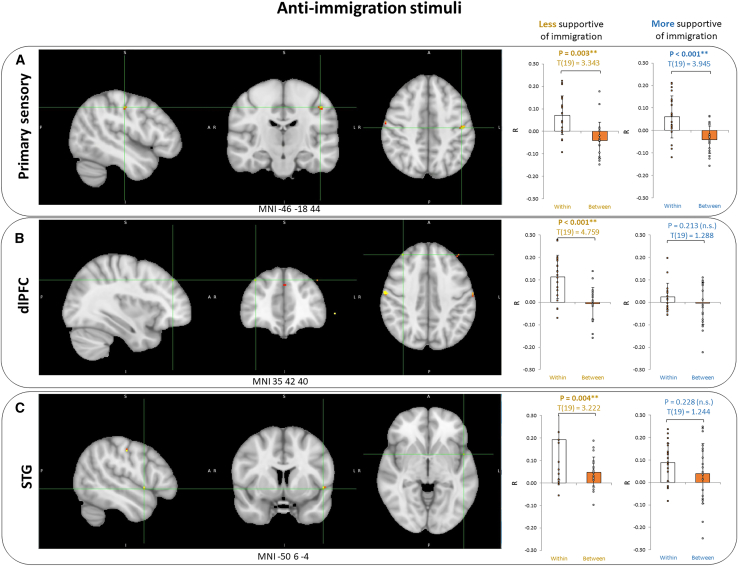
Anti-immigration stimuli Within-between contrast peaks in the (A) Primary sensory, the (B) dlPFC and the (C) STG, in response to anti-immigration stimuli and the contrasts separately in the groups of less immigration supportive and more immigration supportive participants. Data are represented as mean ± SD. Asterisks indicate statistical significance of *p* < 0.001 for the within-between contrast.

The neural within-between group contrasts for anti-immigration stimuli (full map of R value differences in NeuroVault https://identifiers.org/neurovault.image:867873) were slightly different (Figure 3): the peak contrast was found in the left primary somatosensory cortex (MNI -46 -18 44, d = 0.107, *p* < 0.001), and other significant contrasts appeared in the left superior temporal gyrus, STG (MNI -50 6–4, d = 0.097, *p* = 0.002) and right dlPFC (MNI 35 42 40, d = 0.074, *p* < 0.001). The groups did not differ in the contrasts for primary sensory cortex (t(38) = −0.288, *p* = 0.775) or STG (t(38) = −1.575, *p* = 0.124), but the reactions behind dlPFC were significantly different in the more and less supportive groups (t(38) = −2.648, *p* = 0.012). When investigated separately, the primary somatosensory cortex peak was significant in both groups (more supportive t(19) = 3.945, *p* < 0.001, less supportive t(19) = 3.343, *p* = 0.003), the dlPFC contrast only significant for the less supportive group (t(19) = 4.759, *p* < 0.001 vs. t(19) = 1.288, *p* = 0.213), and the superior temporal gyrus contrast also only significant for the less supportive group (t(19) = 3.222, *p* = 0.004 vs. t(19) = 1.244, *p* = 0.228). Interestingly, for the anti-immigration peak contrast, we find an average negative between-group inter-subject correlation, which is significantly different from the average positive between-group inter-subject correlation in the peaks of pro-immigration (t(78) = 2.916, *p* = 0.004) and all stimuli (t(78) = 2.265, *p* = 0.026). When we pooled the neural within-between activation values of the five peaks extracted from pro-immigration and anti-immigration narratives, we found a significant between-groups difference (t(38) = −2.464, *p* = 0.018, Cohen’s d = 0.779) with the less supportive participants showing stronger neural polarization (average 0.117, 95% CI [0.085, 0.149]) than the more supportive participants (average 0.066, 95% CI [0.037, 0.096]). Interestingly, this group difference was not significant (t(38) = −1.102, *p* = 0.318) when pooling only the pro-peaks (Figure 2) but driven (t(38) = −2.477, *p* = 0.018) by the anti-peaks (Figure 3), thereby suggesting that the less supportive participants had stronger neural polarization when processing anti-immigration statements.

We additionally contrasted the peaks extracted from pro-immigration and anti-immigration narratives across the two narrative types and found that the neural responses were significantly different depending on the narrative types in two of the five individual peaks (two extracted from pro-immigration narratives and three from anti-immigration narratives). The significant differences emerged from one of the dlPFC peaks (MNI -28 41 43, t(39) = 3.525, q = 0.005) and the primary sensory cortex peak (MNI -46 -18 44, t(39) = −2.962, q = 0.013). The results of these comparisons can be found in Table S4. With this, we can reject the second null-hypothesis and note that the pro- and anti-immigration stimuli influence the previous results. There was no interaction effect between the content type and the immigration support for these two peaks (for dlPFC, F(1,38) = 0.008, *p* = 0.927, and for primary sensory, F(1,38) = 0.378, *p* = 0.542).

### Correlations across neural and explicit measures

For our third hypothesis, we tested whether the individual neural polarization (within-between group correlation) scores at any peak would be correlated to any self-reported measure, but there were no significant (FDR-corrected) correlations (Pearson q > 0.811 and Spearman q > 0.921, all correlations shown in Tables S5 and S6). However, the pooled score across the five pro- and anti-immigration narrative peaks was significantly correlated with IAS (R = −0.317∗, *p* = 0.046). Although this association did not survive FDR correction (q = 0.812), the uncorrected correlation is in line with the above findings that less support in immigration (i.e., lower IAS) is associated with stronger neural polarization.

## Discussion

We set out to (1) find brain region(s) that reflect the difference between the two groups of mostly immigration-supportive participants. We examined (2) whether the type of narratives (pro- or anti-immigration) influence these differences. Last, we investigated (3) whether these neural differences are connected to attitudinal self-reports.

We outline three important findings: first, we found evidence of neural markers related to immigration support not only at the higher-order frontal regions, but also in sensory and motor regions. Second, we discovered that this divergence is mostly independent of political immigration attitudes. Finally, the narrative type (pro- or anti-immigration) significantly affected the neural responses and group differences and revealed one area where polarization was selective to one of the two immigration support groups.

Relating to our first hypothesis and comparing the neural activations in the two groups of participants, we came to our first main finding: our results showed significant differences between participants that were more and less supportive of immigration in premotor cortex and dorsolateral prefrontal cortex, reflecting both motor and more complex cognitive processes (Figure 2). Earlier studies have come to conflicting results on whether neural polarization origins from higher-order or lower-order areas. The recent similar study by Leong et al. reported low overall intersubject correlation in the motor cortex but found the only significant difference (within-groups ISC bigger than between-groups ISC) between conservatives and liberals in the left dmPFC, also a higher-order brain area.^21^ De Bruin et al. came to similar results as Leong, reporting a significant dmPFC difference between political parties in response to immigration-related narratives.^19^ Katabi et al. however showed ideology-dependent differences also in sensory, motor, and somatosensory regions in addition to the higher-order areas (dlPFC, for one), similarly to our study.^20^ Some earlier studies also highlight differences in neural processing as early as sensory level.^16^^,^^17^^,^^18^ Our study adds to this debate and support the theory that political perspectives affect neural processing also in the lower-order areas.

Importantly, as our second important finding relating to the first hypothesis, although we saw that the left premotor cortex contrast was only significant in the more supportive group and the dlPFC contrast in the less supportive group, we did not find statistically significant differences in neural processing between the immigration support groups in response to all stimuli (Figure S1 shows the values separately in two groups). In Leong et al., the dmPFC within-between groups contrast was driven equally by both groups, meaning they saw ideological symmetry in the polarization.^21^ Similarly, de Bruin et al. reported no partisan asymmetry in the significant neural state differences between Democrats and Republicans that they found.^19^ While Katabi et al.^20^ in their rich results also showed a motor cortex activation only for the rightists group (during a right-wing politician’s speech), they analyzed all the stimuli types separately and identified many differing regions for all types, making their findings complex. For example, rightists displayed a higher dlPFC activation during the processing of campaign ads, but leftists during a left-wing politician’s speech. Our study design allows us to make conclusions about the overall effects of responses to political narratives not depending on political perspectives, adding a new level of confidence and clarity to earlier findings.

Investigating whether the neural responses differ as a function of the stimuli type in response to our second hypothesis, our study design allowed us to expand beyond earlier findings and directly compare the responses to pro- and anti-immigration stimuli, because we used balanced audio statements with similar length and semantic structure (just varying in the immigration support level of the content), which to our knowledge has not been done before. Here, we came to our third important finding and discovered that pro-immigration stimuli elicit the spatial results we could also see on the all-stimuli level: within-between group contrasts in (this time, right) premotor cortex and dlPFC. There were no significant differences between the two support groups while processing the pro-immigration narratives, although, possibly due to reduced statistical power, the right premotor cortex contrast was only significant in the less immigration supportive group, whereas the dlPFC contrast was significant in both support groups (Figure 2). The responses that Katabi et al.^20^ had to a left-wing campaign ad and speech confirm the involvement of dlPFC in both political groups, but they did not find a premotor cortex activation. However, they did see it in rightist responses to the right-wing content, which could mean that the premotor cortex activates when processing narratives close to earlier beliefs and one’s own political ideology.

The neural responses to anti-immigration narratives we found were even more surprising: there were two new significant contrast areas: left primary somatosensory cortex, superior temporal gyrus, with the dlPFC area still showing up, although the location of the peak inside the area varied. Here, we found a significant between-groups contrast in the neural polarization marker in dlPFC, whereas there were no significant differences in the temporal and sensory peaks. Katabi et al. found temporal processing in leftists but not rightists, and synchronized sensory responses within participants that shared strong political opinions in both ideological groups.^20^ We directly compared the neural activations of the extracted peaks in response to pro- and anti-immigration stimuli and found two significant contrasts: in the primary sensory cortex and in dlPFC. Moreover, when pooling over all five peaks extracted in response to anti- and pro-immigration attitudes, there was a significant between-groups difference with the less supportive groups displaying more neural polarization, and this was found only while processing the anti-immigration narratives. Our research further confirms that in some regions, groups with different political attitudes react differently to political content, and adds that it’s extremely important to consider the content type in the analysis, as the neural responses can be significantly different depending on the content type.

Focusing on certain polarizing issues, such as immigration, can increase also general affective and ideological polarization.^3^ The recent review points out that when trying to bring about societal change, ideological asymmetries in polarization should be taken into account: liberal leftists are more willing to push for change, whereas conservative rightists protect the traditions more.^3^ Some notions that might have earlier been liberal have now become conservative, so also these attitudes and labels are not constant in time. At the same time, the review notes that more research is needed to link individual and group differences to outcomes such as polarization.^3^ Earlier research is undecided whether political polarization is symmetrical or not.^25^^,^^26^^,^^27^ The large cohort meta-analysis by Ditto et al. noted a robust symmetry in partisan bias in the USA,^26^ but was criticized by a biased selection of criteria by Baron and Jost.^25^ Kluge et al. reported asymmetry in political polarization between leftists and rightists in Israel on both behavioral and neural levels.^27^ Zebarjadi et al. came to similar asymmetric results while investigating pain empathy,^28^ expanding the ideological asymmetry beyond immediate intergroup attitudes. Our study investigates groups with different immigration support levels; thus we cannot directly weigh into the question of ideological symmetry. We mostly do not find statistically significant between-group differences in the separate polarized responses to political narratives, but when pooling across all peaks activating in response to pro- or anti-immigration narratives, we find a significant between-group difference with the less supportive group displaying higher polarization. In addition, we report one asymmetrical activation in dlPFC in response to anti-immigration narratives – already within a mostly immigration-positive sample.

In this study we find that at the level of brain activity, ideological narratives are processed in a polarized way even when self-reported ideological perspectives slightly differ. This neural finding is in line with other neuroimaging studies on polarization and conflict reporting that the entrenched belief of each side that the other side is wrong is translated as neural activity hindering the ability to reach a common ground and develop constructive dialogue.^29^^,^^30^ Novel psychological approaches were recently proposed to tackle this challenge; for instance the metacognitive capacity for insight into the reliability and fallibility of our own knowledge^31^ or the processing of paradoxical messages about the other side.^32^ Hence such novel strategies are important to bear in mind as tools for reducing polarization facilitating scientifically informed and respectful debates between people who diverge in their ideological beliefs.

### Limitations of the study

Several limitations of the current research should be noted in the interpretation of the results. First, the cohort of participants was overall supportive of immigration – simply with varying levels. Half of the participants reported being leftists and 22.5% rightists, which reflects that both political camps are fairly immigration-positive in Finland. Relatedly, the more supportive group was more uniform in its support whereas in the less supportive group, variability in support was larger, and it may also have been interesting to use a regression approach, which does not use grouping into two subgroups.^22^ Yet, our approach leaned on the group splitting strategy,^19^^,^^20^^,^^21^ which revealed for the first time the concept of neural polarization, and although here the cohort is more ideologically moderate, this in itself is also a strength and complements the findings from those previous studies.^19^^,^^20^^,^^21^ Furthermore, the immigration attitudes were strongly associated with traditional and real-world measures of intergroup attitudes and affect (Figure 1C) as advocated in other recent intergroup studies.^33^ Even more importantly, the neuroimaging results of examining this sample reveal outstanding expressions of neural polarization even between groups with minor deviations in political immigration attitudes. It is also important to note that not all anti-immigration attitudes might be reported due to social norms.^34^ Second, one cannot rule out that the difference in ideological views did not necessarily drive neural polarization, but vice versa. Yet, although the two sub-groups significantly differed (q < 0.02; FDR-corrected) on multiple ideological scales, the present study can not test for a causal relationship between self-reported ideology and its polarized neural representations. Relatedly, it may be argued that there is a dependency between the IAS, which motivated the group splitting, and the following fMRI analyses, which were conducted on data listening to the immigration narratives that are scored for the IAS scale. Despite the relevance of that limitation, one should bear in mind that the IAS were rated after listening to the narratives, whereas the neural data itself is on the perception and processing of the narratives, not on the following IAS moments, which required decision making.

Finally, we further reflect on the sample characteristics in this study. As noted, the two sub-samples only differed on ideological measures, not on other measured characteristics. Yet, there are other aspects to bear in mind in relation to the sample and its possible implications on interpreting the phenomenon of neural polarization. We therefore compared the sample of the present study with that of previous studies reporting neural polarization^19^^,^^20^^,^^21^ – on gender balance and size. The m-f gender balance was of 60-40, 56-44, and 61-39, respectively, thereby reflecting a male preponderance, compared to 25–75 with a clear female preponderance in the current study. As for size, the present and previous studies – all used a similar sample size of approximately 40 individuals. Although none of the studies reported any interaction or effect with gender, and all of them used *a priori* justified sample sizes, it cannot be ruled out that gender or other individual characteristics may affect the phenomenon of neural polarization. Furthermore, sample characteristics in those studies (as well as in most of human neuroscience research) primarily rely on Western, Educated, Industrialized, Rich, and Democratic (WEIRD) individuals, and very often – university students.^35^ It is therefore advisable in future studies to consider increasing sample size and diversity, when constraints allow, and test for possible covariates to gain a more comprehensive outlook of the novel phenomenon of neural polarization, while including the detection of smaller and nuanced effects with increased statistical power and enhanced reliability. Altogether, those limitations should be kept in mind, and future studies would further contribute to expanding this emerging timely domain of investigation by testing whether the inclusion of a more diverse and polarized cohort would result in similar findings as reported here.

## Resource availability

### Lead contact

Further information should be directed to the lead contact, Jonathan Levy, Ph.D. (jonathan.levy@aalto.fi).

### Materials availability

This study did not generate new unique reagents.

### Data and code availability

• Finnish data protection laws do not allow for fMRI data to be made publicly available, as the data cannot be fully anonymized. The individual behavioral data cannot be made publicly available due to restrictions stemming from the ethics permit behind this submission and GDPR. Data can however be shared with scientific collaborators after an amendment to the research ethics permit via Aalto University’s ethics committee and a data transfer agreement.
• Code followed the (available online) pipeline of Leong and colleagues al (Leong et al., 2020) (For further detail cf. SI, fMRIprep pipeline, Data S3).
• Statistical maps of R-values are uploaded to NeuroVault.

## Acknowledgments

This work was supported by the Research Council of Finland, Academy Research Fellow grants to JL (328674 and 352670), a 10.13039/501100003125Finnish Cultural Foundation grant to AK (00220494), and Aalto Brain Center. We acknowledge the computational resources provided by the Aalto Science-IT project. Figure 1 has been designed using assets by macrovector/Freepik.com.

## Author contributions

Conceptualization, N.Z., A.K., M.T., I.P.J., I.J.L., and J.L.; methodology, N.Z., A.K., E.G., and J.L.; investigation and data curation, N.Z., and A.K.; formal analysis, N.Z., and A.K.; writing – N.Z., A.K., and J.L.; writing – review and editing, N.Z., A.K., E.G., M.T., I.P.J., I.J.L., and J.L.

## Declaration of interests

The authors declare no competing interests.

## STAR★Methods

### Key resources table

**Table:** 

REAGENT or RESOURCE	SOURCE	IDENTIFIER
**Software and algorithms**		

*dcm2niix* toolbox (v1.0.20211006)	–	–
*pydeface* (v2.0.2)	–	–
*fMRIprep* v22.1.0	Esteban et al.^36^	–
MATLAB 2023B (MathWorks)	–	–
NifTI toolbox	–	–
IBM SPSS Statistics 28	–	–
Python 3	–	–

### Experimental model and study participant details

Forty-eight healthy Finnish native adolescents and adults participated in a fMRI study. After data preprocessing, 5 participants were excluded from further analysis due to excessive head movement (more than 25% of framewise displacement values > 0.5mm), and 3 more participants were excluded due to excessive noise in their function MRI data, so our final pool was 40 participants (75% female, 19.3 ± 1.6 years of age, 57.5% politically leftist, 20% centrist, 22.5% rightist). Prior to participation in the study, the participants read an information sheet and a privacy notice and signed the participation confirmation form, all approved by the Aalto University Research Ethics Committee. The participants provided their written informed consent to participate in this study. All experiments were performed in accordance with the instructions by the Finnish Advisory Board on Research Integrity (TENK) and General Data Protection Regulation (GDPR).

Our sample size was conventional for fMRI studies, comparable with the previous studies in the same domain (de Bruin and colleagues screened 44, Katabi and colleagues screened 34, while Leong and colleagues screened 36 people), and allowed the detection of effect sizes of at least 0.45 for the full sample and 0.91 between the two groups of 20 with 80% power.^37^

#### Experimental task

Participants were scanned using fMRI (Figure 1A) as they listened to 44 statements (total duration 30 minutes) on immigration to Finland: 22 pro-immigration and 22 anti-immigration (All experimental material are detailed in the SI, Data S2). The randomized order of statements was fixed across participants. For randomization, the order of statements in both stimuli type (pro- and anti-immigration) arrays was separately randomized using a random sequence generator. Next, the mixing order of statement types was manually generated with a maximum of 2 consecutive same-type statements. Finally, the two random arrays were joined. The average length of statements was 16.3s, there was no significant difference between pro-immigration and anti-immigration statement lengths (paired t-test t(21) = -1.265, two-sided p = .22). The statements were balanced to reflect similar issues from a liberal and conservative point of view. For example, a pro-immigration statement would read: “When moving to a new country, migrants bring with them all their knowledge and their skills – they want to be economically productive. They tend to be useful to the Finnish society and their value is bigger than the costs for the public sector” while the corresponding anti-immigration statement was “Muslim immigrants do not bring their economic productivity with them when moving to Finland and they are likely to incur more expenditure than revenue for the public sector”. 54% of anti-immigration and 36% of pro-immigration statements targeted specifically Muslim immigrants, the rest concerned immigration in general. All statements are brought out in SI (Data S2). After each statement, participants were asked to rate the trueness of it on a scale from 1 (completely untrue) to 5 (completely true). In summary, the experiment in the fMRI scanner followed the following order: statement audio presented, 1.5 second break, trueness rating question presented until response button pressed, 16-17 second (randomized length) break for button press wash-out, next statement audio, etc.

After the fMRI measurement, participants answered a collection of attitude questionnaires (c.f., Explicit measures in SI, Data S1).

### Method details

#### Preregistration

The study was preregistered^24^ (https://osf.io/9kjzy) after data collection but before any functional analysis had been conducted. The preregistration includes the general aim and hypotheses of the study, the number of participants, the measured variables (neural activation, agreement with presented narratives, and self-reported attitudes: political inclination, emotions, dehumanisation, feeling thermometer, warmth and competence, perceived threat, right-wing authoritarianism, multiculturalism, resistance to change, group superiority, and workplace attitudes). The preregistration notes the method for the neural data analysis following the procedures described in Leong et al.,^21^ the statistical principles of two-tailed t-tests and Pearson correlation, and data exclusion criteria regarding head movement.

#### Order of presenting the statements

pro2, anti1, anti4, pro18, pro8, anti10, anti21, pro19, pro6, anti9, pro16, anti16, anti8, pro10, anti19, pro13, anti2, pro14, anti6, pro7, pro12, anti15, pro20, anti22, anti18, pro4, pro22, anti12, pro1, anti3, pro5, anti14, pro17, anti7, anti17, pro3, anti13, pro15, anti5, pro21, anti11, pro9, pro11, anti20.

#### Explicit measures

After the fMRI measurement, participants answered a collection of attitude questionnaires: a) self-reported political inclination (Likert-type scale ranging from 1 – extremely rightist to 7 – extremely leftist); b) differently-valenced emotions towards Muslim immigrants: (b1) empathic emotions (3 items) and (b2) negative emotions (5 items) (both a Likert-type scale ranging from 1 – not at all to 7 – very much)^38^; c) dehumanisation (Likert-type scale ranging from 0 – I don’t see Muslims as human at all to 10 – I see Muslims as very much human)^39^; d) feeling thermometer (Likert-type scale ranging from 0 – I have very cold feelings towards Muslim immigrations to 10 – I have very warm feelings towards Muslim immigrants)^40^; e) perceived (e1) warmth and (e2) competence of Muslim immigrants (2 separate items, both a Likert-type scale ranging from 1 – not at all to 5 – very much)^41^; f) perceived threat from Muslim immigrants (4 items, Likert-type scale ranging from 1 – strongly disagree to 7 – strongly agree)^42^; g) Right-Wing Authoritarianism (RWA) (7 items, Likert-type scale from 1 – strongly disagree to 7 – strongly agree, some items reversed)^43^; h) multiculturalism (10 items, Likert-type scale from 1 – strongly disagree to 7 – strongly agree, some items reversed)^44^^,^^45^; i) resistance to change (7 items, Likert-type scale from 1 – strongly disagree to 7 – strongly agree, some items reversed)^46^; j) group superiority scale (8 items, Likert-type scale from 1 – strongly oppose to 7 – strongly favour, some items reversed)^47^; k) discriminatory attitudes against Muslims in workplaces scale (10 items, Likert-type scale from 1 – strongly disagree to 7 – strongly agree, some items reversed with higher scores reflecting more discriminatory attitudes).^48^^,^^49^ All scales were translated into Finnish and modified to best fit the local immigration context. All scales reported in supplemental information.

#### Scale reliability

We tested the reliability of the multi-item explicit scales and calculated Cronbach’s alphas for b) empathic emotions: 3 items, Cronbach’s alpha = .841, negative emotions: 5 items, Cronbach’s alpha = .639; f) perceived threat from Muslim immigrants: 4 items, Cronbach’s alpha = .623; g) RWA :7 items, Cronbach’s alpha = .340; h) multiculturalism: 10 items, Cronbach’s alpha = .834; i) resistance to change: 7 items, Cronbach’s alpha = .639; j) group superiority scale: 8 items, Cronbach’s alpha = .785; k) attitudes towards Muslims in workplaces scale: 10 items, Cronbach’s alpha = .826. Noteworthily, the multi-item scales that produced no significant correlations with IAS were also less reliable (Cronbach’s alpha < .640).

### Quantification and statistical analysis

#### Participant division

We divided the participants into two groups (n = 20 in both) based on their immigration attitudes, similarly to Leong et al.^21^ The groups did not differ (tested with independent samples t-tests) in age (t(38) = -.636, p = .529), gender (t(38) = .370, p = .714) or average framewise displacement (t(38) = -.781, p = .440). For each participant, we calculated the average rating of agreement with pro-immigration and anti-immigration statements, separately. The Immigration Attitude Score (IAS) was calculated by subtracting the mean anti-immigration rating from the mean pro-immigration rating, resulting on a scale from -4 (strongly oppose immigration) to +4 (strongly support immigration). We labelled the participants as more supportive and less supportive (of immigration) based on those scores with the split being the median IAS score (2.7, Figure 1B). The average score in the more supportive group was 3.30 and in the less supportive group 1.58, and almost all participants (37 out of 40) reported immigration positive attitudes with varying strength.

#### fMRI data acquisition and preprocessing

It is noteworthy that all fMRI data analyses followed the (available online) pipeline of Leong and colleagues al^21^ (For further detail c.f. SI, fMRIprep pipeline, Data S3).

Data were collected using a 3 T Siemens Magnetom Skyra MRI scanner and a 30-channel receiving head coil at the Advanced Magnetic Imaging Centre in Aalto University. T2∗-weighted echo-planar imaging (EPI) sequence was used in transverse orientation (TR=1260ms, 40 interleaved slices with 3 mm thickness, TE=32ms) for the functional recording and subsequently the structural MRI scan was acquired using a high-resolution T1-weighted Magnetization Prepared Rapid Gradient Echo (MPRAGE) pulse sequence in sagittal orientation (TR=2530 ms, TE =3.3ms, 176 slices with 1.5 mm thickness).

#### Intersubject correlation analyses

Following the method of Leong et al.,^21^ we concatenated the neural data for the 44 statements (removing the inter-statement data) for each participant. We computed the one-to-average intersubject correlation for the whole sample and calculated the Pearson correlation between the activity time course of a voxel and the average activity time course at the same voxel, repeated this for all voxels and averaged over participants for a map of average R-values. We assessed statistical significance with a nonparametric permutation test, namely, we computed the t-statistic testing for each voxel with the average R-value greater than zero. We flipped the R-value sign for a random subset of participants and recomputed t 10 000 times to generate a null distribution. The p-value was the proportion of the null distribution that was more positive than the original t-statistic. The statistical map was thresholded for voxels that survive correction for multiple comparisons (q < 0.05) to control for false discovery rate (FDR) using the two-stage Benjamini, Krieger, and Yekutieli procedure,^50^ see Leong et al. for further details.^21^

We divided the participants into two groups as described before and searched for voxels with more similar activity time course within-group than between-groups, as also done by Leong and colleagues (2020). For that, we computed the within-group ISC as the voxel-wise ISC between the activity of each participant and the average activity of all other participants in that group. The between-group ISC, accordingly, was calculated as the voxel-wise ISC between the activity of each participant and the average activity of all participants in the other group. We then calculated the difference between the within-group ISC and between-group ISC for each participant and each voxel, as also described by Leong et al. (2020). We averaged this difference across all participants and assessed the significance of the differences with nonparametric permutation testing and FDR correction, similarly as before.^21^ In Figures 2 and 3, statistical significance of those tests at the p level below 0.001 are indicated with Asterisks.

We defined the peak voxels where activity was more similar within groups than between groups and calculated the average activity in each peak separately for more immigration-supportive and less immigration-supportive participants. We obtained the difference between the average activity of the two groups (d) as a neural polarization measure.

#### fMRIprep pipeline

We followed the pipeline published by Leong et al.^21^ on the statements perception data points. Data were transformed to NifTI format using *dcm2niix* toolbox (v1.0.20211006), organized using BIDS format, and then anonymized with *pydeface* (v2.0.2). Next, data were preprocessed using *fMRIprep* v22.1.0^36^ Results included in this manuscript come from preprocessing performed using *fMRIPrep* following the example of Castello et al.^51^ The preprocessing steps (including brain mask and tissue segmentation, spatial normalization, and ICA-AROMA) are described below. Continuing, we denoised the data using a custom Python script (https://version.aalto.fi/gitlab/klugea1/fmri-statements/) and regressed the following nuisance parameters: six motion parameters and their derivatives, global signal, framewise displacement,^52^ the first six noise components estimated by *aCompCor*,^53^ and polynomial trends up to second order.^51^ Additionally, the data were high pass (0.01Hz) filtered.

We continued the analysis following the methods of Leong et al.^21^ The preprocessed data were loaded into MATLAB 2023B (MathWorks) using the NifTI toolbox and normalized by z-scoring across time to remove baseline differences for each statement separately. More detail on the fMRIPrep procedures can be found in https://fmriprep.org/en/20.2.0/citing.html, or alternatively in the SI (Data S3).

#### Behavioral correlation analyses

All self-reported and behavioral material are detailed in the SI (Data S1). We tested for relationships between the neural marker, behavioral IAS scores, and self-reported explicit scores using linear regression and correlations in IBM SPSS Statistics 28.^54^ For explicit scale correlations, we corrected for multiple comparisons by false discovery rate (FDR) procedure^55^ and set the significance threshold at q < 0.05.

#### Differences based on narrative type

To see whether the differences between political groups vary depending on the type of stimuli, we repeated the intersubject correlation analysis using only pro-immigration and only anti-immigration statements, respectively. We then evaluated the within-between contrast between the more and less immigration-supportive participants similarly as done before with the full data. We conducted behavioral correlation analyses similarly as before.
