# Supplementary material for: Polarized neural responses to political narratives are sensitive to small variations in self-reported political perspectives
Source: iScience. 2025 Nov 27;29(1):114268. doi: 10.1016/j.isci.2025.114268 (PMC12794419; doi:10.1016/j.isci.2025.114268)
Supplement: Document S1. Figure S1, Tables S1–S6, and Data S1–S3 [file mmc1.pdf]

iScience, Volume *29*

Supplemental information

Polarized neural responses to political narratives are sensitive to small variations in self-reported political perspectives

Niloufar Zebarjadi, Annika Kluge, Enrico Glerean, Matilde Tassinari, Iiro P. Jääskeläinen, Inga Jasinskaja-Lahti, and Jonathan Levy

<u>Supplementary information</u>

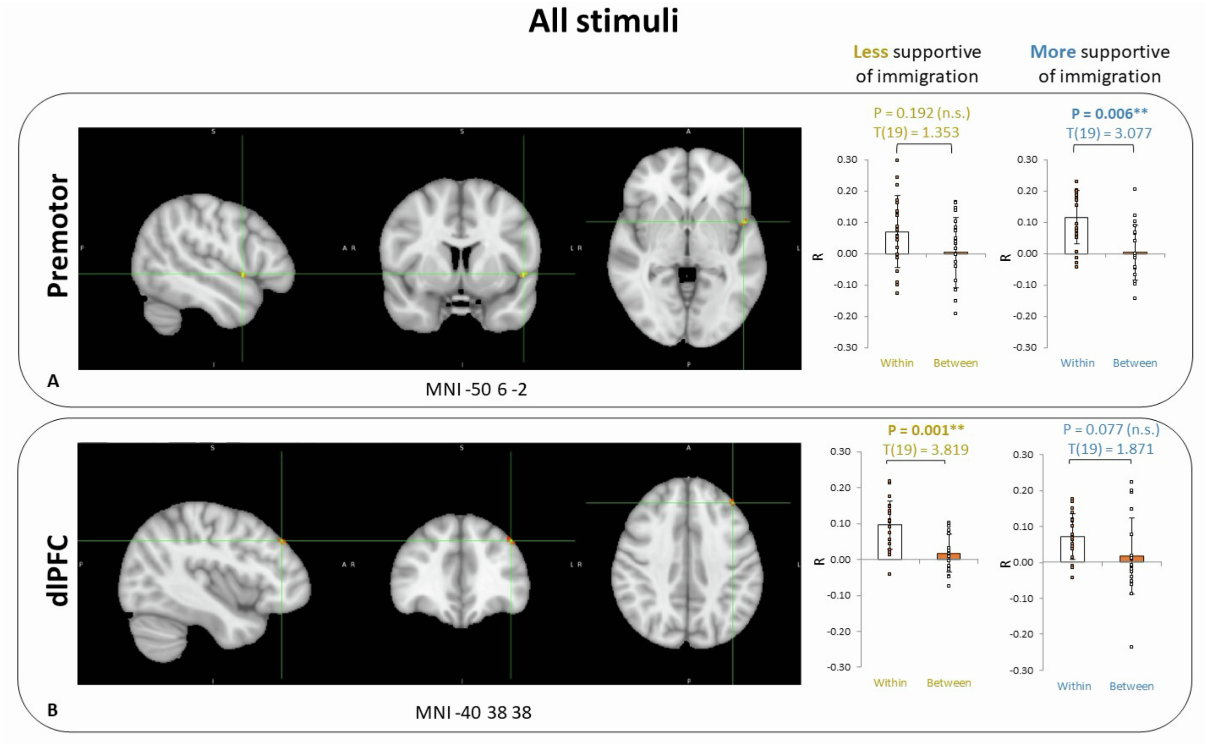

Supplementary Figure 1. Within-between contrast peaks in response to all stimuli and the contrasts separately in the groups of less immigration supportive and more immigration supportive participants. Data are represented as mean +/- SD.

Supplementary Table 1. Explicit attitudes in the two groups. Significant group differences accented with green.

Variable	Less supportive group average	More supportive group average	Between-groups t-test (FDR-corrected p-values reported as q)
a. Political view	4.4, 95% CI [3.82, 4.88]	4.8, 95% CI [4.20, 5.40]	t(38) = 1.175, q = .251, Cohen's d = .372
b1. Empathic emotions	4.28, 95% CI [3.629, 4.937]	4.98, 95% CI [4.467, 5.501]	t(38) = 1.759, q = .162, Cohen's d = .556
b2. Negative emotions	1.54, 95% CI [1.236, 1.844]	1.33, 95% CI [1.142, 1.518]	t(38) = -1.230, q = .251, Cohen's d = -.389
c. Dehumanisation	9.0, 95% CI [8.41, 9.59]	9.4, 95% CI [8.99, 9.81]	t(38) = 1.165, q = .251, Cohen's d = .368
d. Feeling thermometer	6.6, 95% CI [5.99, 7.21]	7.2, 95% CI [6.40, 8.00]	t(38) = 1.247, q = .251, Cohen's d = .394
e1. Warmth	3.9, 95% CI [3.50, 4.20]	4.2, 95% CI [3.87, 4.53]	t(38) = 1.535, q = .216, Cohen's d = .485
e2. Competence	3.4, 95% CI [3.05, 3.75]	4.2, 95% CI [3.87, 4.53]	t(38) = 3.487, q = .004, Cohen's d = .103
f. Perceived threat	1.59, 95% CI [1.329, 1.846]	1.29, 95% CI [1.097, 1.478]	t(38) = -1.955, q = .126, Cohen's d = -.618
g. RWA	1.74, 95% CI [1.552, 1.934]	1.31, 95% CI [1.145, 1.470]	t(38) = -3.636, q = .004, Cohen's d = -1.150
h. Multiculturalism	5.21, 95% CI [4.762, 5.658]	6.02, 95% CI [5.712, 6.238]	t(38) = 3.119, q = .010, Cohen's d = .986
i. Resistance to change	3.56, 95% CI [3.142, 3.974]	3.27, 95% CI [2.826, 3.629]	t(38) = -1.197, q = .251, Cohen's d = -.379
j. Group superiority	2.15, 95% CI [1.658, 2.637]	1.55, 95% CI [1.265, 1.838]	t(38) = -2.200, q = .088, Cohen's d = -.696
k. Discriminatory workplace attitudes	2.29, 95% CI [1.870, 2.710]	1.54, 95% CI [1.367, 1.713]	t(38) = -3.455, q = .004, Cohen's d = -1.093

Supplementary Table 2. Linear regression model: IAS score's dependance on explicit scales.

Variable	Beta	t	p
(Constant)		1.287	.210
a. Political view	-.277	-1.615	.118
b1. Empathic emotions	.154	1.041	.308
b2. Negative emotions	-.142	-1.156	.258
c. Dehumanisation	.055	.475	.639
d. Feeling thermometer	.271	1.882	.071
e1. Warmth	-.153	-1.146	.262
e2. Competence	-.098	-.652	.520
f. Perceived threat	-.097	-.632	.533
g. RWA	-.208	-1.750	.092
h. Multiculturalism	.306	1.563	.130
i. Resistance to change	.038	.293	.772
j. Group superiority	-.155	-1.004	.325
k. Discriminatory workplace attitudes	-.366	-1.732	.095

Supplementary table 3. Correlations across IAS and explicit scales.

Scales		IAS	a	b1	b2	c	d	e1	e2	f	g	h	i	j
a	R	.321*												
	p	0.044												
b1	R	.539**	.517**											
	p	0.000	0.001											
b2	R	-0.149	0.101	0.094										
	p	0.360	0.537	0.564										
c	R	0.205	0.220	0.071	0.023									
	p	0.205	0.172	0.661	0.890									
d	R	.399*	.430**	.417**	0.109	0.181								
	p	0.011	0.006	0.007	0.503	0.264								
e1	R	0.241	0.271	0.145	0.049	.346*	.413**							
	p	0.135	0.091	0.373	0.763	0.029	0.008							
e2	R	.385*	0.195	0.218	0.223	0.274	.329*	.433**						
	p	0.014	0.229	0.176	0.167	0.087	0.038	0.005						
f	R	-.409**	-.390*	-0.191	0.299	-0.117	0.000	-0.274	-0.217					
	p	0.009	0.013	0.238	0.061	0.472	1.000	0.087	0.178					
g	R	-0.301	-0.274	-0.243	-0.178	-0.143	-0.072	-0.134	-.314*	0.138				
	p	0.060	0.087	0.131	0.271	0.378	0.660	0.409	0.049	0.395				
h	R	.663**	.588**	.486**	0.033	0.214	.404**	.415**	.502**	-.463**	-0.189			
	p	0.000	0.000	0.001	0.839	0.184	0.010	0.008	0.001	0.003	0.243			
i	R	-0.145	-0.139	-0.211	-0.214	-0.019	-0.110	0.012	-0.284	0.116	0.311	-0.074		
	p	0.372	0.391	0.192	0.186	0.906	0.501	0.942	0.075	0.474	0.050	0.651		
j	R	-.547**	-.469**	-.470**	-0.082	-0.277	-0.179	-0.262	-.390*	0.298	0.232	-.623**	0.019	
	p	0.000	0.002	0.002	0.617	0.084	0.269	0.102	0.013	0.062	0.149	0.000	0.910	
k	R	-.743**	-.332*	-.508**	0.050	-0.231	-.330*	-.388*	-.565**	.528**	0.230	-.718**	0.307	.571**
	p	0.000	0.036	0.001	0.760	0.152	0.037	0.013	0.000	0.000	0.153	0.000	0.054	0.000

*. Correlation is significant at the 0.05 level (2-tailed).

**. Correlation is significant at the 0.01 level (2-tailed).

Supplementary Table 4. Comparisons across narrative types between pro-immigration and anti-immigration narrative neural peaks.

**Peak extracted from…**	**Peak**	**t(39)**	**p**
… pro-immigration narratives	MNI 5 12 68 (premotor)	1.656	.106
	MNI -28 41 43 (dlPFC)	3.525	.001
… anti-immigration narratives	MNI -46 -18 44 (primary sensory)	-2.962	.005
	MNI 35 42 40 (dlPFC)	-.541	.592
	MNI -50 6 -4 (STG)	-1.396	.171

Supplementary Table 5. Neural and explicit correlations (Pearson). * reflects the values that were significant before FDR correction.

Pearson correlations (FDR-corrected)		All premotor	All dlPFC	Pro premotor	Pro dlPFC	Anti primary sensory	Anti dlPFC	anti STF	Pooled peak
**IAS**	**R**	0.153	-0.020	-0.275	-0.017	-0.050	-0.302	-0.114	-.317*
	**q**	0.965	0.974	0.952	0.974	0.965	0.812	0.965	0.812
**a**	**R**	0.093	-0.288	0.194	0.154	0.122	-0.100	0.011	0.151
	**q**	0.965	0.884	0.965	0.965	0.965	0.965	0.974	0.965
**b1**	**R**	0.046	-0.131	0.020	0.056	-0.120	-0.167	-0.069	-0.115
	**q**	0.965	0.965	0.974	0.965	0.965	0.965	0.965	0.965
**b2**	**R**	0.204	-0.010	-0.027	-0.024	0.025	0.047	0.104	0.064
	**q**	0.965	0.974	0.974	0.974	0.974	0.965	0.965	0.965
**c**	**R**	-0.126	0.230	-0.106	-0.032	-0.110	-.360*	-0.169	-.314*
	**q**	0.965	0.965	0.965	0.974	0.965	0.812	0.965	0.812
**d**	**R**	0.029	-0.094	0.122	0.100	0.069	-0.136	0.019	0.075
	**q**	0.974	0.965	0.965	0.965	0.965	0.965	0.974	0.965
**e1**	**R**	-0.082	-0.053	-0.054	-0.068	0.196	-0.063	-0.041	-0.012
	**q**	0.965	0.965	0.965	0.965	0.965	0.965	0.965	0.974
**e2**	**R**	0.120	-0.139	-0.185	0.076	0.167	-0.168	-0.063	-0.095
	**q**	0.965	0.965	0.965	0.965	0.965	0.965	0.965	0.965
**f**	**R**	0.048	-0.013	-0.063	-0.115	-0.057	-0.092	0.090	-0.062
	**q**	0.965	0.974	0.965	0.965	0.965	0.965	0.965	0.965
**g**	**R**	0.005	-0.096	0.122	-0.169	-0.220	0.077	.316*	0.130
	**q**	0.987	0.965	0.965	0.965	0.965	0.965	0.812	0.965
**h**	**R**	0.208	-0.227	-0.091	0.126	0.146	-0.250	0.067	-0.001
	**q**	0.965	0.965	0.965	0.965	0.965	0.965	0.965	0.997
**i**	**R**	0.232	0.029	0.072	-.372*	0.064	-0.061	.385*	0.155
	**q**	0.965	0.974	0.965	0.812	0.965	0.965	0.812	0.965
**j**	**R**	-0.009	0.148	-0.042	-0.032	-0.172	0.238	0.105	0.044
	**q**	0.974	0.965	0.965	0.974	0.965	0.965	0.965	0.965
**k**	**R**	0.051	-0.011	0.188	-0.061	0.073	0.219	0.241	0.307
	**q**	0.965	0.974	0.965	0.965	0.965	0.965	0.965	0.812

Supplementary Table 6. Neural and explicit correlations (Spearman). * reflects the values that were significant before FDR correction.

Spearman correlations (FDR-corrected)		All premotor	All dlPFC	Pro premotor	Pro dlPFC	Anti primary sensory	Anti dlPFC	anti STF	pooled peak
**IAS**	**rs**	0.137	-0.076	-0.219	-0.024	-0.137	-0.296	-0.158	-.359*
	**q**	0.922	0.997	0.922	0.997	0.922	0.922	0.922	0.922
**a**	**rs**	0.101	-.355*	0.250	0.225	0.120	-0.128	-0.004	0.170
	**q**	0.995	0.922	0.922	0.922	0.922	0.922	0.997	0.922
**b1**	**rs**	0.020	-0.156	0.121	0.097	-0.137	-0.165	-0.050	-0.105
	**q**	0.997	0.922	0.922	0.995	0.922	0.922	0.997	0.987
**b2**	**rs**	0.222	-0.028	0.016	0.023	-0.028	-0.013	0.122	0.032
	**q**	0.922	0.997	0.997	0.997	0.997	0.997	0.922	0.997
**c**	**rs**	-0.205	0.148	-0.050	-0.009	0.083	-0.127	-0.286	-0.238
	**q**	0.922	0.922	0.997	0.997	0.997	0.922	0.922	0.922
**d**	**rs**	0.038	-0.057	0.120	0.157	0.007	-0.058	-0.001	0.029
	**q**	0.997	0.997	0.922	0.922	0.997	0.997	0.997	0.997
**e1**	**rs**	-0.061	-0.009	-0.015	-0.092	0.054	0.042	-0.044	-0.024
	**q**	0.997	0.997	0.997	0.997	0.997	0.997	0.997	0.997
**e2**	**rs**	0.158	-0.091	-0.144	0.077	0.140	-0.121	-0.088	-0.123
	**q**	0.922	0.997	0.922	0.997	0.922	0.922	0.997	0.922
**f**	**rs**	0.112	0.146	-0.122	-0.240	-0.007	-0.024	0.209	-0.098
	**q**	0.967	0.922	0.922	0.922	0.997	0.997	0.922	0.995
**g**	**rs**	-0.041	-0.035	0.129	-0.162	-0.170	0.134	0.283	0.164
	**q**	0.997	0.997	0.922	0.922	0.922	0.922	0.922	0.922
**h**	**rs**	0.239	-0.259	0.018	0.105	0.069	-0.226	0.086	-0.011
	**q**	0.922	0.922	0.997	0.987	0.997	0.922	0.997	0.997
**i**	**rs**	0.200	0.151	0.011	-.329*	0.006	-0.056	.356*	0.135
	**q**	0.922	0.922	0.997	0.922	0.997	0.997	0.922	0.922
**j**	**rs**	0.046	0.207	-0.083	-0.057	-0.210	0.208	0.145	0.023
	**q**	0.997	0.922	0.997	0.997	0.922	0.922	0.922	0.997
**k**	**rs**	0.010	-0.001	0.035	-0.038	0.127	0.272	0.209	0.273
	**q**	0.997	0.997	0.997	0.997	0.922	0.922	0.922	0.922

Data S1

Surveys (translated from Finnish; see original form in the next section)

a. Political inclination
(1 Extremely left-wing / socialism, ecosocialism – 5 Extremely right-wing / conservatism) In general, ideas associated with the left include a stronger role for the state in economic regulation, social equality, and social justice. The right, on the other hand, emphasizes conservatism, support for the market economy, individual freedom and responsibility, and the importance of national defense.

b. Empathic and negative emotions
(1 Not at all – 7 A lot) When you think about Muslim immigrants, to what extent do you feel the following emotions? • Anger • Hatred • Empathy • Sympathy • Fear • Pity • Guilt • Shame

c. *Dehumanization*
(0 Not at all human – 10 Very human)
Psychological studies have shown that people tend to place different human groups on different levels of perceived humanity. The following scale represents levels of humanity, where 0 means very low humanity and 10 means very high humanity. Please choose the number that best reflects how human you perceive Muslim immigrants to be.

d. *Feeling Thermometer*
(0 Very cold feelings – 10 Very warm feelings) If emotions could be described using a thermometer with a scale from 0 to 10, how would you describe your feelings toward Muslim immigrants living in Finland?

e. Warmth and Competence (1 Not at all – 5 Very much)

How friendly and competent do you consider Muslim immigrants in Finnish society to be?

- Friendly
- Competent

f. Perceived Threat (1 Strongly disagree – 7 Strongly agree)

To what extent do you agree with the following statements?

1. Muslim immigrants pose a threat to Finland's security.
2. Muslim immigrants threaten the Finnish welfare state.
3. Muslim immigrants pose a threat to Finnish society.
4. Muslim immigrants pose a threat to me and my family

g. Right-Wing Authoritarianism (RWA) (1 Strongly disagree – 7 Strongly agree)

Please indicate how much you agree or disagree with the following statements:

- The only way our country can survive the coming crisis is to return to our traditional values, put a group of tough leaders in power, and silence the spreaders of bad ideas.
- Our country needs free thinkers who have the courage to oppose traditional ways, even if it upsets many people.

- Our country will eventually be destroyed if we do not crush the wrong ideas that erode our morals and traditional beliefs.
- Everyone should be allowed to live the lifestyle, religious beliefs, and sexual preferences they choose, even if it makes them different from others.
- This country would run much better if certain troublemaking groups would just shut up and accept their traditional place in society.
- There is no “one right way” to live life; everyone must create their own way.
- “Old-fashioned ways” and “old-fashioned values” still show the best way to live.

h. Multiculturalism (1 Strongly disagree – 7 Strongly agree)

To what extent do you agree or disagree with the following statements regarding different cultures living together in Finland?

- Native Finns should acknowledge that Finnish society consists of diverse ethnic groups.
- Ethnic minorities should be supported in preserving their cultural heritage in Finland.
- From society’s perspective, it would be best if everyone forgot their ethnic and cultural background as quickly as possible.
- A society with diverse ethnic and cultural groups is better able to solve new problems as they arise.
- Finnish national unity is weakened if people of different ethnic and cultural backgrounds stick to their traditions.
- If ethnic minorities want to preserve their culture, they should not display it.
- A highly multicultural society has more problems with national unity than societies with one or two core cultural groups.
- Native Finns should do more to learn about the customs and traditions of the various ethnic and cultural groups in the country.
- Members of ethnic groups should encourage their children to maintain their home country’s culture and traditions.
- People coming to Finland should adapt their behavior to match that of native Finns.

i. Resistance to Change (1 Strongly disagree – 7 Strongly agree)

Please indicate how much you agree or disagree with the following statements:

- I generally view change as a negative thing.
- I prefer doing familiar things over trying new and different ones.
- Changing plans feels very burdensome to me.
- I often change my mind.
- I do not change my mind easily.
- Once I reach a conclusion, I am unlikely to change it.
- My views remain consistent over time.

j. Group Superiority (1 Strongly against – 7 Strongly in favor)

Choose on a scale of 1–7 how much you support or oppose the following statements. You can answer quickly: first impressions are usually best.

- An ideal society requires that some groups are positioned above others.

- Some groups of people are simply inferior to others.
- No group should dominate in society.
- Disadvantaged groups deserve just as much as those in higher positions.
- Group equality should not be our primary goal.
- It is unfair to try to make all groups equal.
- We should do what we can to equalize conditions for different groups.
- We should work to give all groups equal opportunities to succeed.

k. Discriminatory Workplace Attitudes (1 Strongly disagree – 7 Strongly agree)

To what extent do you agree or disagree with the following statements regarding multiculturalism in Finnish workplaces?

- I believe Finns are more capable and competent workers than Muslim immigrants.
- I think Muslim immigrants cause more problems in the workplace than Finns.
- I would feel equally comfortable with a Muslim-background supervisor as with a Finnish one.
- Most Muslim immigrant supervisors do a great job.
- I think Muslim immigrants are less efficient workers than Finns.
- In most cases, I would prefer a supervisor who is not Muslim.
- I know many more competent Finns who should have gotten the job instead of the Muslim who was hired.
- I believe that hiring more Muslim immigrants can only benefit the Finnish labor market.
- I believe that any differences in competence between Muslim immigrants and Finns are only due to the lack of opportunities for immigrants to develop their skills.
- I believe that if Muslim immigrants were given more opportunities and work experience, they would be just as competent as Finnish workers.

Surveys (original language)

f. Political inclination (1 Erittäin vasemmistolainen / sosialismi, ekososialismi – 5 erittäin oikeistolainen / konservatismi)

Yleistäen, vasemmistolaisuuteen liittyviä ideoita ovat esimerkiksi valtion vahvempi rooli talouselämän ohjaamisessa, yhteiskunnallinen tasa-arvo ja sosiaalinen oikeudenmukaisuus. Oikeisto puolestaan korostaa konservatismia, markkinatalouden suosimista, yksilön vapautta ja vastuuta sekä maanpuolustuksen tärkeyttä.

g. Empathic and negative emotions (1 en yhtään -7 paljon)

Kun ajattelet muslimimaahanmuuttajia, missä määrin tunnet seuraavia tunteita?

- Suuttumus
- Viha
- Empatia
- Sympatia
- Pelko
- Sääli

- Syyllisyys
- Häpeä

h. Dehumanisation (0 en lainkaan inhimillisenä – 10 erittäin inhimillisenä)

Psykologiset tutkimukset ovat osoittaneet, että ihmiset ovat taipuvaisia asettamaan eri ihmisryhmiä eri inhimillisyyden tasoille. Seuraava asteikko esittää inhimillisyyden tasoja, joista 0 tarkoittaa hyvin vähäistä inhimillisyyttä ja 10 erittäin korkeaa inhimillisyyttä. Ole hyvä, ja valitse asteikolta sopiva numero, joka kuvaa, kuinka inhimillisinä näet muslimimaahanmuuttajat.

i. Feeling thermometer (0 hyvin kylmiä tunteita – 10 hyvin lämpimiä tunteita)

Jos tunteita voisi kuvata lämpömittarina, jonka asteikko on 0-10, kuinka kuvaisit sen avulla tunteitasi Suomessa asuvia muslimimaahanmuuttajia kohtaan?

j. Warmth and competence (1 en yhtään – 5 erittäin)

Kuinka ystävällisinä ja pätevinä pidät muslimimaahanmuuttajia suomalaisessa yhteiskunnassa?

- Ystävällinen
- Pätevä

k. Perceived threat (1 täysin eri mieltä – 7 täysin samaa mieltä)

Missä määrin olet samaa mieltä seuraavien väittämien kanssa?

- 1.Muslimimaahanmuuttajat uhkaavat Suomen turvallisuutta.
- 2. Muslimimaahanmuuttajat uhkaavat suomalaista hyvinvointivaltiota.
- 3. Muslimimaahanmuuttajat uhkaavat suomalaista yhteiskuntaa.
- 4. Muslimimaahanmuuttajat uhkaavat minua ja perhettäni.

l. RWA (1 täysin samaa mieltä – 7 täysin samaa mieltä)

Ole hyvä ja merkitse, missä määrin olet samaa tai eri mieltä seuraavien väitteiden kanssa.

- Ainoa tapa, jolla maamme voi selviytyä tulevasta kriisistä, on palata perinteisiin arvoihimme, laittaa joukko kovia johtajia valtaan ja hiljentää huonojen ideoiden levittäjät.
- Maamme tarvitsee vapaita ajattelijoita, joilla on rohkeutta vastustaa perinteisiä tapoja, vaikka tämä häiritsisi monia ihmisiä.
- Maamme tuhotaan joskus, jos emme kukista ajatusvääristymiä, jotka syövät moraaliamme ja perinteisiä uskomuksia.
- Jokaisen tulisi saada omata haluamansa elämäntapa, uskonnollinen vakaumus ja seksuaaliset mieltymykset, vaikka ne tekisivätkin hänestä erilaisen suhteessa muihin.
- Tämä maa toimisi paljon paremmin, jos tietyt häirikköryhmät vain hiljentyisivät ja hyväksyisivät ryhmänsä perinteisen paikan yhteiskunnassa.
- Ei ole "yhtä oikeaa tapaa" elää elämää; jokaisen on luotava oma tapansa.
- "Vanhanaikaiset tavat" ja "vanhanaikaiset arvot" osoittavat edelleen parhaan tavan elää.

m. Multiculturalism (1 täysin eri mieltä – 7 täysin samaa mieltä)

Missä määrin olet samaa tai eri mieltä seuraavian väitteiden kanssa, jotka koskevat Suomessa yhdessä eläviä eri kulttuureja?

- Syntyperäisten suomalaisten tulisi myöntää, että suomalainen yhteiskunta koostuu erilaisista etnisistä ryhmistä.
- Etnisiä vähemmistöjä tulisi auttaa säilyttämään kulttuuriperintönsä Suomessa.
- Yhteiskunnan näkökulmasta olisi parasta, jos kaikki ihmiset unohtaisivat etnisen ja kulttuurisen taustansa mahdollisimman nopeasti.
- Yhteiskunta, jossa on erilaisia etnisiä ja kulttuurisia ryhmiä, pystyy paremmin ratkaisemaan uusia ongelmia niiden ilmetessä.
- Suomen kansallinen yhtenäisyys heikentyy, jos eri etnisen ja kulttuurisen taustan omaavat ihmiset pitäytyvät perinteissään.
- Jos etniset vähemmistöt haluavat säilyttää kulttuurinsa, heidän ei pitäisi esitellä sitä.
- Hyvin monikulttuurisella yhteiskunnalla on enemmän ongelmia kansallisen yhtenäisyyden kanssa kuin yhteiskunnilla, joissa on yksi tai kaksi peruskulttuuriryhmää.
- Syntyperäisten suomalaisten tulisi tehdä enemmän oppiakseen maan eri etnisten ja kulttuuriryhmien tapoja ja perinteitä.
- Etnisten ryhmien jäsenten tulisi kannustaa lapsiaan säilyttämään kotimaansa kulttuuri ja perinteet.
- Suomeen tulevien ihmisten tulisi muuttaa käyttäytymistään syntyperäisten suomalaisten käyttäytymisen mukaan.

n. Resistance to Change (1 täysin eri mieltä – 7 täysin samaa mieltä)

Ole hyvä ja merkitse, missä määrin olet samaa tai eri mieltä seuraavien väitteiden kanssa.

- Pidän muutosta yleensä kielteisenä asiana.
- Pidän vanhojen ja tuttujen asioiden tekemisestä sen sijaan, että kokeilisin uusia ja erilaisia.
- Suunnitelmien muuttaminen tuntuu minusta todella vaivalloiselta.
- Muutan usein mieltäni.
- En muuta mieltäni helposti.
- Kun olen saapunut johonkin johtopäätökseen, en todennäköisesti muuta mieltäni.
- Näkemykseni säilyvät ajan mittaan johdonmukaisina.

o. Group superiority (1 täysin vastaan – 7 täysin puolesta)

Valitse asteikolla 1-7, kuinka puolesta tai vastaan olet seuraavia väitteitä. Voit vastata nopeasti: ensivaikutelma on yleensä paras.

- Ihanteellinen yhteiskunta vaatii sen, että jotkut ihmisryhmät ovat korkeammassa asemassa kuin toiset.
- Jotkut ihmisryhmät yksinkertaisesti ovat alempiarvoisempia kuin toiset.
- Minkään ryhmän ei tulisi hallita yhteiskunnassa.
- Huonommassa asemassa olevat ryhmät ansaitsevat yhtä paljon kuin korkeammassa asemassa olevat.
- Ryhmien tasa-arvon ei pitäisi olla ensisijainen tavoitteemme.
- On epäoikeudenmukaista yrittää tehdä ryhmistä tasa-arvoisia.
- Meidän tulisi tehdä voitavamme olosuhteiden tasaamiseksi eri ryhmille.

- Meidän pitäisi työskennellä antaaksemme kaikille ryhmille yhtäläisen mahdollisuuden menestyä.

p. <u>Discriminatory workplace attitudes (1 täysin eri mieltä - 7 täysin samaa mieltä)</u>

Missä määrin olet samaa tai eri mieltä seuraavien väitteiden kanssa, jotka koskevat työympäristöjen monikulttuurisuutta Suomessa?

- Mielestäni suomalaiset ovat kyvykkäämpiä ja pätevämpiä työntekijöitä kuin muslimimaahanmuuttajat.
- Mielestäni muslimimaahanmuuttajat aiheuttavat enemmän vaikeuksia työpaikalla kuin suomalaiset.
- Oloni olisi yhtä luonteva muslimitaustaisen esimiehen kuin suomalaisen esimiehen kanssa.
- Suurin osa muslimimaahanmuuttajista, jotka ovat esimiesasemassa, tekevät loistavaa työtä.
- Mielestäni muslimimaahanmuuttajat ovat vähemmän tehokkaampia työntekijöitä kuin suomalaiset.
- Useimmissa tilanteissa suosisin esimiestä, kuka ei ole muslimi.
- Tiedän paljon enemmän pätevämpiä suomalaisia, joiden olisi kuulunut saada työpaikka verrattuna työhön palkattuun muslimiin.
- Uskon, että palkkaamalla enemmän muslimimaahanmuuttajia voidaan ainoastaan edistää suomalaisia työmarkkinoita.
- Uskon, että mahdolliset erot pätevyydessä muslimimaahanmuuttajien ja suomalaisten välillä johtuvat ainoastaan siitä, ettei maahanmuuttajille ole annettu tarpeeksi mahdollisuuksia kartuttaa kykyjään.
- Uskon, että jos muslimimaahanmuuttajat saisivat enemmän mahdollisuuksia ja työkokemusta, he olisivat aivan yhtä päteviä kuin suomalaiset työntekijät.

Data S2

Statements used in the study with translations

<u>Maahanmuuttajavastaiset /Anti-immigrants</u>

1. Pakolaisten määrän kasvaminen merkitsee korkeampia veroja paikallisväestölle, sillä pakolaiset elävät paikallisten veronmaksajien rahoilla. Verovaroilla tulisi ensisijaisesti auttaa suomalaisia eikä pakolaisia, ja suomalaisilla on oltava etuja verrattuna maahanmuuttajiin.
   More refugees mean higher taxes for the locals as they live on local taxpayers' money. Tax funds should be used primarily by helping Finns and not refugees and locals must have advantages compared to immigrants.

2. Yksin saapuvat, turvapaikkaa hakevat lapset ovat ”ankkurilapsia”. Vanhemmat käyttävät lapsiaan hyväkseen lähettämällä heidät Suomeen yksin, jotta he voivat seurata itse perässä ja hyödyntää edistynyttä sosiaaliturvajärjestelmäämme.
   Unaccompanied asylum-seeking children are "anchor children". Parents take advantage of their children by sending them to Finland alone, so that they could follow suit themselves and take advantage of our advanced social security system.

3. Suurin osa muslimipakolaisista on kouluttamattomia ja lukutaidottomia ihmisiä, jotka eivät käy töissä ja pitävät sosiaaliturvan varassa elämisestä. Esimerkiksi ”turvapaikkashoppailijat” matkustavat läpi Euroopan ja käyttävät hyväkseen eurooppalaista sosiaaliturvaa.
   Most of Muslim refugees are uneducated and illiterate people, who are not employed and like to live solely on social security. For example, "asylum shoppers" travel across Europe and take advantage of the social security system.

4. Muslimipakolaiset alentavat palkkoja ja vaikeuttavat matalasti koulutettujen paikallisten työntekijöiden työnsaantia. Halpaa työvoimaa ei pitäisi suosia, ja paikallisväestö tulisi aina asettaa Suomessa etusijalle. Suomen tulee ensin huolehtia suomalaisista.
   Muslim refugees push down wages and make it more difficult for low-skilled local workers to get jobs. Cheap labor should not be favored, and locals should always be prioritized in Finland. Finland must take care of its own people first.

5. Muslimimaahanmuuttajien työttömyysaste on huomattavasti suurempi kuin paikallisväestön, minkä vuoksi he saavat enemmän sosiaaliturvaetuuksia ja suomalaiset puolestaan vähemmän. Tämä tilanne ei ole oikein ja se tulisi korjata.
   The unemployment rate of Muslim foreigners is considerably higher than locals, so there are more benefits to foreigners, and this results in less benefits for locals; this situation is not right and should be rectified.

6. Muslimimaahanmuuttajat eivät tuo mukanaan taloudellista tehokkuuttaan muuttaessaan Suomeen ja todennäköisemmin aiheuttavat enemmän kustannuksia kuin tuloja julkisella sektorilla.
   Muslim immigrants do not bring their economic productivity with them when moving to Finland and they are likely to incur more expenditure than revenue for the public sector.

7. Muslimipakolaisia ja turvapaikanhakijoita koskeva byrokratia vie liikaa resursseja ja ei ole vaivan arvoista. Suomen tulisi kontrolloida Suomeen saapuvien kouluttamattomien pakolaisten määrää suomalaisten edun ja Suomen menestyksen takia.
   The bureaucracy of handling Muslim refugees and asylum seekers takes too many resources and is not worth the effort; Finland should control the number of low-skilled refugees coming into Finland for the benefit of the locals and the prosperity of the country.

8. Suomi on ottanut liikaa muslimimaahanmuuttajia, mikä on johtanut ongelmiin julkisissa palveluissa, töiden saatavuudessa ja palkoissa. Asumisen tarjoaminen on vaikeaa ja vaatii paljon resursseja, joita ei ole helposti saatavilla.
   Finland has taken too many Muslim immigrants in, which has resulted in problems in public services, job availability and wages. Providing housing is difficult and requires a lot of resources that are not readily available.
9. Suomi tarvitsee korkeasti koulutettuja työntekijöitä ja sen ei pitäisi sallia matalasti koulutettuja pakolaisia saapumasta Suomeen. Haluamme maamme jatkavan kasvuaan, rikastumista ja menestystä, joka voidaan saavuttaa ainoastaan älykkäiden ihmisten avulla.

Finland needs workers with high skill levels and should not allow low skilled refugees to enter Finland; we want our country to continue its growth, richness and prosperity and that can only be done by bright people.

10. Sosioekonomiset ja verotukselliset hyödyt eivät pitkälläkään aikavälillä ylitä pakolaisten kotouttamiseen liittyviä kustannuksia. Pakolaiset ovat ylimääräinen taakka Suomen taloudelle. Se on menetys, jota emme voi kompensoida tai sallia.
    The socio-economic and fiscal benefits do not outweigh the associated refugee integration costs in the long run. Refugees are an extra burden to the economy of Finland. That is a loss that we cannot compensate or allow.

11. Turvapaikanhakijat, jotka saapuvat Suomeen ilman asianmukaisia asiakirjoja ja passia ovat laittomia maahanmuuttajia ja heille ei tulisi antaa minkäänlaisia etuuksia. Näissä tapauksissa henkilöiden tunnistamiseen käytetyt resurssit eivät ole lopputuloksen arvoisia.
    Asylum seekers who arrive in Finland without proper paperwork and passports are illegal immigrants and should not be given any benefits. In these cases, the resources that are spent trying to identify them, are not worth the result.

12. Euroopan rajat vuotavat ja muslimiturvapaikanhakijoiden määrä kasvaa vuosi vuodelta. Tämän vuoksi meidän hyvinvointimme ja turvallisuutemme ovat uhattuna. Eurooppa on toiminut holtittomasti, ja sillä on vaikeuksia myöntää tekemänsä virheet.
    European borders are leaking – there are more Muslim asylum seekers every year. For this reason, our wellbeing and safety have been threatened. Europe has acted recklessly and has trouble admitting its own mistakes.

13. Terrorismirikokset Suomessa ovat suoraa seurausta harkitsemattomasta maahanmuuttopolitiikasta. Muslimimaahanmuuttajien kanssa ei ole mahdollista keskustella järkevästi, minkä vuoksi Suomi tarvitsee vahvistettuja poliisivoimia ylläpitämään järjestystä. Lainrikkojia tulisi rangaista ankarammin.
    Terrorist crimes in Finland are a direct result of reckless immigration policy. It is not possible to reason with Muslim immigrants – that's why Finland needs stronger police forces to keep the order. The lawbreakers should be punished more severely.

14. Radikaali islam ja pakolaiset ovat Suomen akuutein sisäisen turvallisuuden uhka. Siksi entistä harvemman muslimimaahanmuuttajan tulisi sallia asettuvan maahan. Paikallisväestön tulisi saada säilyttää turvallisuudentunteensa.

Islamic radicalism and refugees are Finland's most acute internal security threat. Therefore, fewer Muslim refugees entering Finland should be allowed to settle in the country. The locals must be allowed to feel safe.

15. Muslimimaahanmuuttajilla tulisi olla Suomessa omat asuinalueensa. Heidän ei pitäisi sekoittua paikallisväestöön, sillä heidän rikollisuusasteensa on korkeampi ja he ovat epäilyttävämpiä. Antamalla heidän elää yhdessä paikallisväestön kanssa saatetaan uhata nyky-yhteiskuntaa ja vaikuttaa kielteisellä tavalla sen tulevaisuuteen.
    Muslim immigrants in Finland should have their own separated region to live in; they should not mix with the locals, because they have a higher criminal rate and are more suspicious. Letting them live together with locals can threaten the society and negatively influence it in the future.

16. Muslimimaahanmuuttajien kouluttamisessa tai kotouttamisessa suomalaiseen yhteiskuntaan ei ole järkeä, koska he eivät halua oppia paikallisia tapoja ja pitävät tiukasti kiinni omasta kielestään ja kulttuuristaan. Muslimimaahanmuuttajalapset ovat oppimistuloksissa 2-3 vuotta jäljessä suomalaislapsia.
    There is no point in trying to educate or integrate Muslim immigrants into Finnish society, because they do not want to learn the local ways and try very hard to keep on to their own languages and customs. Muslim immigrant children are 2-3 years behind Finnish children in their study results.

17. Meidän tulisi nähdä vaivaa turvapaikanhakijoiden vuosittaisen lukumäärän vähentämiseksi. Suomalaisen paikallisväestön osuus tulisi olla ainakin 90 % Suomen koko populaatiosta. Emme voi antaa suomalaisen kansallisuuden lähestyä sukupuuton uhkaa.
    We should make efforts to lessen the yearly number of asylum seekers – Finnish locals must always make up at least 90% of the whole population of Finland. We cannot let the Finnish nationality get anywhere close to the threat of extinction.

18. Turvapaikanhakijoiden tulisi saada asua Suomessa vain rajoitetun ajan, minkä jälkeen heille tulisi tarjota lähtöpaketti Suomesta poistumiseen. Suomen tulee tunnistaa auttamisen rajansa, ja vaikka voimme hyväksyä vierailijat, emme suhtaudu myönteisesti loisiin.
    Asylum seekers should be allowed to stay in Finland only for a limited amount of time. Finland should provide a starter-package for asylum seekers leaving Finland

after that limited time. Finland must know its boundaries and while we accept visitors, we do not tolerate parasites.

19. Taloudellisista syistä maahan pyrkiviä ja pakolaisia tulisi kohdella eri tavalla. Suomen tulisi rajoittaa maahanmuuttoa mahdollisimman paljon ja asettaa erittäin tiukat vaatimukset ihmisille, jotka haluavat muuttaa tänne asumaan.
    Economic migrants should be regarded and treated differently compared to refugees. Immigration to Finland should be restricted as much as possible – there should be very strict requirements for people wishing to live here.

20. Turvapaikanhakijoiden ja muiden ulkomaalaisten pääsyä pysyvään oleskelulupaan tulisi Suomessa selvästi rajoittaa. Kaikkien ehdokkaiden on todella osoitettava olevansa arvokkaita yhteiskunnalle, ja pysyvän oleskeluluvan myöntäminen tulisi olla pikemminkin poikkeus kuin sääntö.
    Access to the permanent residence permit for asylum seekers and refugees in Finland must be clearly restricted. Any candidate must really prove to be valuable to the society and the acceptance should be more of an exception than a rule.

21. Suurin osa pakolaisista on nuoria ja terveitä miehiä. Heidän pitäisi olla taistelemassa kotimaassaan parempien elinolojen puolesta, jotta meillä ei olisi niin suurta ongelmaa maahanmuuttajien määrän kanssa. Suomessa he ovat levottomia ja aiheuttavat ongelmia.
    Most refugees are young, healthy men. They should be fighting in their home country to make the conditions there livable, so we wouldn't have such a big issue with the abundance of immigrants. In Finland they are restless and cause trouble.

22. Hyväksymällä enemmän muslimimaahanmuuttajia Suomeen luodaan jakoa eri mielipiteitä omaavien paikallisten välillä ja paljon tarpeettomia jännitteitä rauhallisessa yhteiskunnassamme. Tämän vuoksi muslimimaahanmuuttajat eivät hyödytä tai edistä yhteiskuntaamme.
    Accepting more Muslim immigrants in Finland creates a division among locals and a lot of unnecessary tensions in our peaceful society. This is why Muslim immigrants are not benefiting nor contributing to our society.

<u>Maahanmuuttajamyönteiset / Pro-immigrants</u>

1. Pakolaiset tulevat maksamaan veroja yhtä lailla kuin paikallisetkin. Kaikkien yhteiskunnassa toimivien tulisi omata samanlaiset oikeudet ja velvollisuudet verojen maksamisen ja sosiaaliturvaetuuksien saamisen suhteen. Ulkomaalaiset antavat osuutensa suomalaiselle yhteiskunnalle samalla tavalla kuin paikallisväestökin. *Refugees will become taxpayers just like locals in Finland. Everyone functioning as part of this society should have similar rights and responsibilities when it comes to paying taxes and using funds from our social security system. Foreigners contribute to the Finnish society the same way locals do.*

2. Suurimmassa avuntarpeessa ovat yksin saapuvat, turvapaikkaa hakevat lapset. Kuka tahansa vanhempi haluaisi olla lapsensa kanssa ja lapsi vanhempansa. Kyseisiä lapsia tulisi kohdella suurella huolenpidolla. *The people in the biggest need are unaccompanied asylum-seeking children. Any parent would want to be with their child and every child wishes to be with their parents. These children must be treated with care.*

3. Monet muslimipakolaiset ovat koulutettuja ja osaavia ihmisiä, jotka voisivat työllään suuresti edistää Suomen taloutta. Pakolaiset haluavat yleensä työskennellä, ja Suomen toimenpiteet järjestelmän väärinkäyttäjien tavoittamiseksi ovat tehokkaita. *Many Muslim refugees are skilled and knowledgeable people who could greatly contribute to Finland's economy. Refugees usually want to work, and Finland's measures to catch people misusing the system are effective.*

4. Muslimipakolaiset voivat tuoda enemmän kilpailua ja tarjontaa työmarkkinoille. He voivat vakauttaa palkkoja. Työnantajien on myös taloudellisesti kannattavaa harkita kansainvälisen työvoiman palkkaamista, sillä he saattavat olla halukkaita tekemään töitä, joita paikallisväestö ei halua tehdä. *Muslim refugees offer more competition and selection in the job market. They can help stabilize the wages. It is profitable also for employers to consider buying in international workforce, who may be willing to do the jobs locals do not want to do.*

5. Paikallisväestö on epäreilussa etulyöntiasemassa työmarkkinoilla, minkä seurauksena muslimimaahanmuuttajien työttömyysaste on korkeampi. Jos voimme ratkaista tämän työnsaantiin liittyvän vinouman, niin valtion sosiaaliturvaetuudet jakautuisivat tasapainoisemmin niitä eniten tarvitseville.

Locals have unfair advantages in the job market in Finland. That results in higher unemployment rates of Muslim immigrants. If we can solve the bias in employment, the benefits from the country can be shared fairly and equally to people in the biggest need.

6. Maahanmuuttajat tuovat mukanaan kaiken tietonsa ja taitonsa muuttaessaan uuteen maahan - he haluavat tehdä osuutensa talouden hyväksi. He ovat yleensä hyödyksi suomalaiselle yhteiskunnalle, ja heidän arvonsa on suurempi kuin julkiselle sektorille aiheutuvat kulut.
   When moving to a new country, migrants bring with them all their knowledge and their skills – they want to be economically productive. They tend to be useful to the Finnish society and their value is bigger than the costs for the public sector.

7. Pakolaiset ja turvapaikanhakijat luovat uusia työpaikkoja paikallisväestölle saapuessaan tänne – he tarvitsevat asiakirjoja ja muuta apua. Lisäksi pitkällä aikavälillä maahanmuuttajat tuovat suomalaiselle yhteiskunnalle enemmän arvoa kuin kuormitusta.
   Refugees and asylum seekers create new jobs for locals already by coming here – they need documentation and help. Besides, in the long run, immigrants provide more value to the community than stress.

8. Suomi on ollut hyvin vastuullinen ja hyvä roolimalli vastaanottaessaan EU-maahanmuuttajia. Palvelut ja työmarkkinat ovat selvinneet kuormituksesta hyvin. Paikallisväestö voi työllistyä tarjoamalla asumista ja muita tukipalveluita maahanmuuttajille. Suomalaiset työnantajat voivat löytää uusia työntekijöitä ja kasvattaa liiketoimintaansa.
   Finland has been very responsible and a good role model for letting in EU immigrants. The services and the job market have coped well with the stress. Locals can also find employment providing housing and support services for the immigrants. Finnish employers can find new workforce and grow their business.

9. Suomessa on monia vapaita työpaikkoja eri koulutustaustan omaaville työntekijöille. Työnhakijoiden runsaus voi avata monia uusia ja kiinnostavia liiketoimintamahdollisuuksia. Täten ei tule tuomita tänne saapuvia ihmisiä heidän taitotasonsa perusteella.

There are very many jobs available in Finland for workers of all skill levels. An abundance of work force could open many new and exciting business opportunities. Therefore, it is important not to judge people coming here based on their skill level.

10. Pakolaisten kotouttamisesta aiheutuvat kustannukset ovat vähäpätöisiä verrattuna pitkän aikavälin valtiontaloudellisiin hyötyihin. Asumisen ja töiden tarjoaminen pakolaisille antaa myös mahdollisuuden vahvistaa paikallista taloutta.
The cost incurred by the integration of refugees is negligible compared to the long-term benefits for the economy in Finland. Providing refugees with housing and work is also an opportunity to boost the local economy.

11. Turvapaikanhakijat, jotka saapuvat ilman asianmukaisia asiakirjoja ovat suuren avun tarpeessa olevia ihmisiä. Meidän on oltava luovia todistaaksemme heidän henkilöllisyytensä ja historiansa. Meidän tulisi kohdella kaikkia ihmisiä kunnioituksella ja auttaa heitä parhain mahdollisin tavoin.
Asylum seekers arriving without proper paperwork are people in utmost need for help. We must be creative in helping them prove their identity and history. We should treat all people with respect and try to help them in the best way possible.

12. Eurooppa tekee kelvollista työtä rajavalvonnan kanssa. Muslimiturvapaikanhakijoiden määrän kasvaessa järjestelmän on oltava huolella harkittu, mutta ihmisten lukumäärä itsessään ei ole syynä rikollisuuteen ja köyhyyteen yhteiskunnassamme.
Europe is doing an alright job with border control. With the growing number of Muslim asylum seekers, the system must be well thought through, but the number of people itself isn't a reason for crime and poverty in our society.

13. Terrorismirikokset Suomessa ovat seurausta muslimimaahanmuuttajien huonosta kohtelusta. Suomen tarvitsee olla vastaanottavaisempi ja kotouttaa tulokkaat paremmin yhteiskuntaan. Vain jos muslimimaahanmuuttajat eivät tunne oloaan turvalliseksi, voivat he alkaa käyttäytyä tavoin, joita ei yleisesti suvaita.
Terrorist crimes in Finland are a result of poor handling of Muslim immigrants. Finland needs to be more welcoming and better at integrating newcomers into society. Only if Muslim immigrants do not feel safe, they can start to behave in ways not commonly tolerated.

14. Radikaali islam on turvallisuusuhka ympäri maailman. Muslimipakolaiset ja radikaalin islamin toisiinsa rinnastavista mielleyhtymistä on luovuttava, sillä näitä

kahta ryhmää ei voida pitää yhtenä ja samana. Muslimipakolaisten on annettava asettua maahan Suomeen kuulumisen tunteen luomiseksi.
Islamic radicalism is a security threat all around the world. The link between Muslim refugees and Islamic radicalism must be broken as these two groups cannot be considered as one. Muslim refugees must be allowed to settle in the country to create a sense of belonging.

15. Muslimimaahanmuuttajien tulisi asua samoilla alueilla paikallisväestön kanssa kaikissa maan eri osissa. Tämä voisi olla tehokkain tapa toimia yhtenä yhteiskuntana, kun taas heidän erottaminen omille asuinalueilleen aiheuttaisi vain enemmän ongelmia ja erkaantumista.
    Muslim immigrants should live in the same regions as locals and be present in all parts of the country. This could be the most effective way to function as one society, whereas separating them would only cause more issues and differentiation.

16. Muslimimaahanmuuttajat ovat halukkaita oppimaan suomen kielen ja sopeutumaan paikalliseen kulttuuriin, joten hallituksen tulisi suunnitella ympäristö, joka helpottaisi heidän koulutustaan ja kotoutumistaan. Heidän lapsensa voivat tuoda paikallisväestölle opettavaisia uusia kulttuurisia tapoja ja taitoja.
    Muslim immigrants are willing to learn Finnish language and adapt to the local culture, so government should design a setting that facilitates their education and integration into society. Their children can bring new culture and skills from which natives can learn the good things.

17. Turvapaikanhakijoiden vuosittainen lukumäärä saa kasvaa, eikä ulkomaalaisten prosenttiosuuden kasvu Suomessa ole ongelma. Suomi tarvitsee ulkomaalaisia työntekijöitä ja yhteiskunnallista monimuotoisuutta yhteiskunnallisen kasvun vuoksi.
    The yearly number of asylum seekers can grow, and it is not a problem if the percentage of foreigners in Finland increases. Finland needs foreign workers and societal diversity to grow as a society.

18. Turvapaikanhakijoiden olisi annettava kotoutua ja osallistua suomalaiseen yhteiskuntaan ja tulla osaksi sitä, minkä lisäksi heitä tulisi kohdella tasavertaisesti paikallisväestön kanssa. Jos paikallisväestö kohtelee heitä kunnioituksella, voimme odottaa samaa myös vastineeksi.

Asylum seekers should be allowed to integrate, contribute and become part of the Finnish society and be treated equally to locals. If locals treat them with respect, we can expect the same in return.

19. Taloudellisista syistä maahan pyrkiviä ja pakolaisia tulisi kohdella samalla tavalla. Suomen tulisi jatkossakin suhtautua tulokkaisiin vastaanottavaisesti ja olla eniten ensikertalaisten turvapaikanhakijoiden määrää korottavien maiden joukossa Euroopassa.
    Economic migrants (”migrating because of economic reasons”) and refugees should be treated the same way. Finland should continue to be welcoming toward newcomers and be among the countries in Europe that are increasing the number of first-time asylum seekers the most.
20. Suomeen saapuvia ihmisiä tulisi kannustaa jäämään ja tukea heitä suomalaiseen yhteiskuntaan kotoutumisessa. Vain ajan kanssa voi yhteiskunta todella muuttua heterogeeniseksi ja sen kaikki jäsenet sopeutua yhteen jaettuun kulttuuriin.
    People coming to Finland should be encouraged to stay and supported for a positive integration in the Finnish society. Only with time can society really become more heterogenous and all its members adapt to one shared culture.

21. Suomessa väestö vanhenee, mikä kuormittaa paljon sosiaaliturvajärjestelmää ja työmarkkinoita. Pakolaiset voivat olla hyödyllistä työvoimaa ja auttaa tasapainon ylläpitämisessä.
    In Finland the population is aging, and that is putting a lot of pressure of social security mechanisms and employment market. Refugees can be a useful workforce and help in maintaining the balance.

22. Ihmiskunnan tulisi keskittyä enemmän toistensa auttamiseen ja yhdessä toimimiseen, ja vähemmän tiettyjen rajojen ja ihmisryhmien, kuten maahanmuuttajien ja suomalaisten, välisten erojen ylläpitämiseen. Lopulta olemme kaikki kuitenkin ihmisiä.
    Humankind should be more focused on helping one another and functioning together, and less focused on maintaining certain limits and differences between groups of people, for example, between immigrants and native Finns. In the big picture, we are all human beings.

Data S3

fMRIPrep's documentation

Results included in this manuscript come from preprocessing performed using fMRIPrep 22.1.0+0.gce344b39.dirty (Esteban, Markiewicz, et al. 2018; Esteban, Blair, et al., 2018) (RRID:SCR_016216), which is based on Nipype 1.8.5 (K. Gorgolewski et al. (2011); K. J. Gorgolewski et al. (2018); RRID:SCR_002502).

Anatomical data preprocessing

A total of 1 T1-weighted (T1w) images were found within the input BIDS dataset. The T1-weighted (T1w) image was corrected for intensity non-uniformity (INU) with N4BiasFieldCorrection (Tustison et al. 2010), distributed with ANTs 2.3.3 (Avants et al. 2008, RRID:SCR_004757), and used as T1w-reference throughout the workflow. The T1w-reference was then skull-stripped with a Nipype implementation of the antsBrainExtraction.sh workflow (from ANTs), using OASIS30ANTs as target template. Brain tissue segmentation of cerebrospinal fluid (CSF), white-matter (WM) and gray-matter (GM) was performed on the brain-extracted T1w using fast (FSL 6.0.5.1:57b01774, RRID:SCR_002823, Zhang, Brady, and Smith 2001). Volume-based spatial normalization to two standard spaces (MNI152NLin6Asym, MNI152NLin2009cAsym) was performed through nonlinear registration with antsRegistration (ANTs 2.3.3), using brain-extracted versions of both T1w reference and the T1w template. The following templates were selected for spatial normalization: FSL's MNI ICBM 152 non-linear 6th Generation Asymmetric Average Brain Stereotaxic Registration Model [Evans et al. (2012), RRID:SCR_002823; TemplateFlow ID: MNI152NLin6Asym], ICBM 152 Nonlinear Asymmetrical template version 2009c [Fonov et al. (2009), RRID:SCR_008796; TemplateFlow ID: MNI152NLin2009cAsym].

Functional data preprocessing

For each of the 1 BOLD runs found per subject (across all tasks and sessions), the following preprocessing was performed. First, a reference volume and its skull-stripped version were generated using a custom methodology of fMRIPrep. Head-motion parameters with respect to the BOLD reference (transformation matrices, and six corresponding rotation and translation parameters) are estimated before any spatiotemporal filtering using mcflirt (FSL 6.0.5.1:57b01774, Jenkinson et al. 2002). BOLD runs were slice-time corrected to 0.589s (0.5 of slice acquisition range 0s-1.18s) using 3dTshift from AFNI (Cox and Hyde 1997, RRID:SCR_005927). The BOLD time-series (including slice-timing correction when applied) were resampled onto their original, native space by applying the transforms to correct for head-motion. These resampled BOLD time-series will be referred to as preprocessed BOLD in original space, or just preprocessed BOLD. The BOLD reference was then co-registered to the T1w reference using mri_coreg (FreeSurfer) followed by flirt (FSL 6.0.5.1:57b01774, Jenkinson and Smith 2001) with the boundary-based registration (Greve and Fischl 2009) cost-function. Co-registration was configured with six degrees of freedom. Several confounding time-series were calculated based on the preprocessed BOLD: framewise displacement (FD), DVARS and three region-wise global signals. FD was computed using two formulations following Power (absolute sum of relative motions, Power et al. (2014)) and Jenkinson (relative root mean square displacement between affines, Jenkinson et al. (2002)). FD and DVARS are calculated for each functional run, both using their implementations in Nipype (following the definitions by Power et al. 2014). The three global signals are extracted within the CSF, the WM, and the whole-brain masks. Additionally, a set of physiological regressors were extracted to allow for component-based noise correction (CompCor, Behzadi et al. 2007). Principal components are estimated after high-pass filtering the preprocessed BOLD time-series (using a discrete cosine filter with 128s cut-off) for the two CompCor variants: temporal (tCompCor) and anatomical

(aCompCor). tCompCor components are then calculated from the top 2% variable voxels within the brain mask. For aCompCor, three probabilistic masks (CSF, WM and combined CSF+WM) are generated in anatomical space. The implementation differs from that of Behzadi et al. in that instead of eroding the masks by 2 pixels on BOLD space, a mask of pixels that likely contain a volume fraction of GM is subtracted from the aCompCor masks. This mask is obtained by thresholding the corresponding partial volume map at 0.05, and it ensures components are not extracted from voxels containing a minimal fraction of GM. Finally, these masks are resampled into BOLD space and binarized by thresholding at 0.99 (as in the original implementation). Components are also calculated separately within the WM and CSF masks. For each CompCor decomposition, the k components with the largest singular values are retained, such that the retained components' time series are sufficient to explain 50 percent of variance across the nuisance mask (CSF, WM, combined, or temporal). The remaining components are dropped from consideration. The head-motion estimates calculated in the correction step were also placed within the corresponding confounds file. The confound time series derived from head motion estimates and global signals were expanded with the inclusion of temporal derivatives and quadratic terms for each (Satterthwaite et al. 2013). Frames that exceeded a threshold of 0.5 mm FD or 1.5 standardized DVARS were annotated as motion outliers. Additional nuisance timeseries are calculated by means of principal components analysis of the signal found within a thin band (crown) of voxels around the edge of the brain, as proposed by (Patriat, Reynolds, and Birn 2017). The BOLD time-series were resampled into standard space, generating a preprocessed BOLD run in MNI152NLin6Asym space. First, a reference volume and its skull-stripped version were generated using a custom methodology of fMRIPrep. Automatic removal of motion artifacts using independent component analysis (ICA-AROMA, Pruim et al. 2015) was performed on the preprocessed BOLD on MNI space time-series after removal of non-

steady state volumes and spatial smoothing with an isotropic, Gaussian kernel of 6mm FWHM (full-width half-maximum). Corresponding "non-aggresively" denoised runs were produced after such smoothing. Additionally, the "aggressive" noise-regressors were collected and placed in the corresponding confounds file. All resamplings can be performed with a single interpolation step by composing all the pertinent transformations (i.e. head-motion transform matrices, susceptibility distortion correction when available, and co-registrations to anatomical and output spaces). Gridded (volumetric) resamplings were performed using antsApplyTransforms (ANTs), configured with Lanczos interpolation to minimize the smoothing effects of other kernels (Lanczos 1964). Non-gridded (surface) resamplings were performed using mri_vol2surf (FreeSurfer).

Many internal operations of fMRIPrep use Nilearn 0.9.1 (Abraham et al. 2014, RRID:SCR_001362), mostly within the functional processing workflow. For more details of the pipeline, see the section corresponding to workflows in fMRIPrep's documentation.

fMRIprep references

Abraham, Alexandre, Fabian Pedregosa, Michael Eickenberg, Philippe Gervais, Andreas Mueller, Jean Kossaifi, Alexandre Gramfort, Bertrand Thirion, and Gael Varoquaux. 2014. "Machine Learning for Neuroimaging with Scikit-Learn." *Frontiers in Neuroinformatics* 8. https://doi.org/10.3389/fninf.2014.00014.

Avants, B. B., C. L. Epstein, M. Grossman, and J. C. Gee. 2008. "Symmetric Diffeomorphic Image Registration with Cross-Correlation: Evaluating Automated Labeling of Elderly and Neurodegenerative Brain." *Medical Image Analysis* 12 (1): 26–41. https://doi.org/10.1016/j.media.2007.06.004.

Behzadi, Yashar, Khaled Restom, Joy Liau, and Thomas T. Liu. 2007. "A Component Based Noise Correction Method (CompCor) for BOLD and Perfusion Based fMRI." *NeuroImage* 37 (1): 90–101. https://doi.org/10.1016/j.neuroimage.2007.04.042.

Cox, Robert W., and James S. Hyde. 1997. "Software Tools for Analysis and Visualization of fMRI Data." *NMR in Biomedicine* 10 (4-5): 171–78. https://doi.org/10.1002/(SICI)1099-1492(199706/08)10:4/5<171::AID-NBM453>3.0.CO;2-L.

Esteban, Oscar, Ross Blair, Christopher J. Markiewicz, Shoshana L. Berleant, Craig Moodie, Feilong Ma, Ayse Ilkay Isik, et al. 2018. "fMRIPrep 22.1.0+0.gce344b39.dirty." *Software*. https://doi.org/10.5281/zenodo.852659.

Esteban, Oscar, Christopher Markiewicz, Ross W Blair, Craig Moodie, Ayse Ilkay Isik, Asier Erramuzpe Aliaga, James Kent, et al. 2018. "fMRIPrep: A Robust Preprocessing Pipeline for Functional MRI." *Nature Methods*. https://doi.org/10.1038/s41592-018-0235-4.

Evans, AC, AL Janke, DL Collins, and S Baillet. 2012. "Brain Templates and Atlases." *NeuroImage* 62 (2): 911–22. https://doi.org/10.1016/j.neuroimage.2012.01.024.

Fonov, VS, AC Evans, RC McKinstry, CR Almli, and DL Collins. 2009. "Unbiased Nonlinear Average Age-Appropriate Brain Templates from Birth to Adulthood." *NeuroImage* 47, Supplement 1: S102. https://doi.org/10.1016/S1053-8119(09)70884-5.

Gorgolewski, K., C. D. Burns, C. Madison, D. Clark, Y. O. Halchenko, M. L. Waskom, and S. Ghosh. 2011. "Nipype: A Flexible, Lightweight and Extensible Neuroimaging Data Processing Framework in Python." *Frontiers in Neuroinformatics* 5: 13. https://doi.org/10.3389/fninf.2011.00013.

Gorgolewski, Krzysztof J., Oscar Esteban, Christopher J. Markiewicz, Erik Ziegler, David Gage Ellis, Michael Philipp Notter, Dorota Jarecka, et al. 2018. "Nipype." *Software*. https://doi.org/10.5281/zenodo.596855.

Greve, Douglas N, and Bruce Fischl. 2009. "Accurate and Robust Brain Image Alignment Using Boundary-Based Registration." *NeuroImage* 48 (1): 63–72. https://doi.org/10.1016/j.neuroimage.2009.06.060.

Jenkinson, Mark, Peter Bannister, Michael Brady, and Stephen Smith. 2002. "Improved Optimization for the Robust and Accurate Linear Registration and Motion Correction of Brain Images." *NeuroImage* 17 (2): 825–41. https://doi.org/10.1006/nimg.2002.1132.

Jenkinson, Mark, and Stephen Smith. 2001. "A Global Optimisation Method for Robust Affine Registration of Brain Images." *Medical Image Analysis* 5 (2): 143–56. https://doi.org/10.1016/S1361-8415(01)00036-6.

Lanczos, C. 1964. "Evaluation of Noisy Data." *Journal of the Society for Industrial and Applied Mathematics Series B Numerical Analysis* 1 (1): 76–85. https://doi.org/10.1137/0701007.

Patriat, Rémi, Richard C. Reynolds, and Rasmus M. Birn. 2017. "An Improved Model of Motion-Related Signal Changes in fMRI." *NeuroImage* 144, Part A (January): 74–82. https://doi.org/10.1016/j.neuroimage.2016.08.051.

Power, Jonathan D., Anish Mitra, Timothy O. Laumann, Abraham Z. Snyder, Bradley L. Schlaggar, and Steven E. Petersen. 2014. "Methods to Detect, Characterize, and Remove Motion Artifact in Resting State fMRI." *NeuroImage* 84 (Supplement C): 320–41. https://doi.org/10.1016/j.neuroimage.2013.08.048.

Pruim, Raimon H. R., Maarten Mennes, Daan van Rooij, Alberto Llera, Jan K. Buitelaar, and Christian F. Beckmann. 2015. "ICA-AROMA: A Robust ICA-Based Strategy for Removing

Motion Artifacts from fMRI Data." *NeuroImage* 112 (Supplement C): 267–77. https://doi.org/10.1016/j.neuroimage.2015.02.064.

Satterthwaite, Theodore D., Mark A. Elliott, Raphael T. Gerraty, Kosha Ruparel, James Loughead, Monica E. Calkins, Simon B. Eickhoff, et al. 2013. "An improved framework for confound regression and filtering for control of motion artifact in the preprocessing of resting-state functional connectivity data." *NeuroImage* 64 (1): 240–56. https://doi.org/10.1016/j.neuroimage.2012.08.052.

Tustison, N. J., B. B. Avants, P. A. Cook, Y. Zheng, A. Egan, P. A. Yushkevich, and J. C. Gee. 2010. "N4itk: Improved N3 Bias Correction." *IEEE Transactions on Medical Imaging* 29 (6): 1310–20. https://doi.org/10.1109/TMI.2010.2046908.

Zhang, Y., M. Brady, and S. Smith. 2001. "Segmentation of Brain MR Images Through a Hidden Markov Random Field Model and the Expectation-Maximization Algorithm." *IEEE Transactions on Medical Imaging* 20 (1): 45–57. https://doi.org/10.1109/42.906424.
